# Potential genetic association between coffee/caffeine consumption and erectile dysfunction: a Mendelian randomization study and meta-analysis

**DOI:** 10.3389/fendo.2024.1400491

**Published:** 2024-10-09

**Authors:** Nana Xiang, Yanhua Hu, Wenchun Peng, Mei Luo, Hong Chen, Qiuhua Zhang

**Affiliations:** ^1^ Department of Urology, The Affiliated Nanchong Central Hospital of North Sichuan Medical College (University), Nanchong, Sichuan, China; ^2^ Department of Urology, Nanchong Central Hospital (Nanchong Clinical Research Center), Nanchong, Sichuan, China

**Keywords:** erectile dysfunction, coffee/caffeine consumption, causal association, Mendelian randomization, meta-analysis

## Abstract

**Background:**

Coffee is a widely consumed beverage with potential benefits for various chronic diseases. Its effect on reducing erectile dysfunction (ED) risk is unclear. This Mendelian randomization (MR) study investigates the impact of coffee/caffeine consumption on ED.

**Methods:**

Two sets of coffee consumption-associated genetic variants at the genome-wide significance level were obtained from recent studies of coffee consumption. Taking into account other sources of caffeine, genetic variants associated with caffeine consumption from tea were also obtained. The inverse variance weighted (IVW) method was utilized as the primary analysis. Sensitivity analysis methods and meta-analysis methods were performed to confirm the robustness of the results, while the genetic variants associated with confounders, e.g., diabetes and hypertension, were excluded.

**Results:**

Genetically predicted coffee/caffeine consumption was unlikely to be associated with the risk of ED in the Bovijn datasets, with similar directional associations observed in the FinnGen datasets. The combined odds ratio for ED was 1.011 (95% CI 0.841–1.216, *p*=0.906) for coffee consumption from the genome-wide meta-analysis, 1.049 (95% CI 0.487–2.260, *p*=0.903) for coffee consumption from the genome-wide association study, and 1.061 (95% CI 0.682–1.651, *p*=0.793) for caffeine from tea.

**Conclusion:**

Using genetic data, this study found no association between coffee/caffeine consumption and the risk of ED.

## Introduction

Erectile dysfunction (ED) is the inability of male individuals to achieve or maintain an erection sufficient for satisfactory sexual intercourse for more than 3 months (1). This is a common male condition, seriously affecting mental health and quality of life (2). The largest European multicenter population-based study has claimed that the average prevalence of ED among European men aged 40 to 79 was 30%, and increased with age (3). Some modifiable risk factors, such as diabetes, cardiovascular diseases, hypertension, obesity, and depression, have been suggested to increase the risk of ED (4, 5).

There is limited understanding of other factors that might have a potential benefit on ED, such as coffee intake. Coffee, easily available in daily life and one of the most widely consumed beverages in the world, is a rich source of antioxidants and anti-inflammatory compounds (6). Caffeine is the most abundant component in coffee and also exists in tea (7). The hypothesis behind the consumption of coffee in ameliorating erectile function is the fact that ED is often a precursor of impending cardiovascular risk, which is reduced after the use of antioxidants (8). However, some existing studies have reported contradictory results (6, 9). As traditional retrospective studies often involve confounding factors, more high-quality studies are needed to investigate the relationship.

Mendelian randomization (MR) is an innovative epidemiological approach that explores the causal relationship between exposure factors and outcome risk by using single-nucleotide polymorphisms (SNPs) as genetic variants to mimic randomization in randomized controlled trials (10). As is well known, genotype randomization occurs after conception and is less affected by acquired confounding factors. Thus, natural randomization and avoiding the interference of reverse causality and confounding factors are unique advantages of MR analysis (11).

To the best of our knowledge, no previous study has investigated the causal associations between coffee/caffeine consumption and ED by using MR analysis. In this study, we used an MR design to investigate the possible effect of coffee/caffeine consumption on ED.

## Methods

### Study design

**Figure 1:**
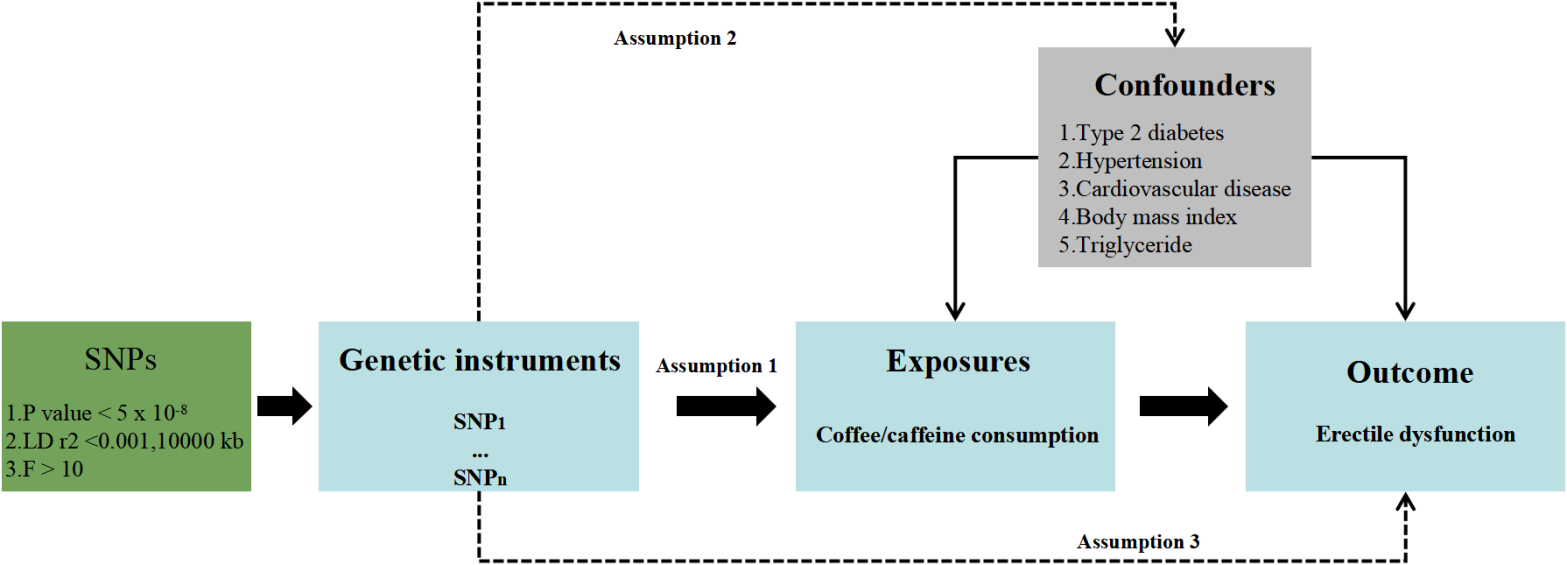
Study design overview.

An overview of the MR study design is described in **Figure 1**. To make causal estimations obtained from the two-sample MR analysis valid, three critical assumptions must be met: (1) SNPs should associate with the risk factor of interest (the relevance assumption); (2) SNPs should not be impacted by confounders of the risk factor–outcome association (the independence assumption); and (3) SNPs should affect the outcome solely via the risk factor (the exclusion restriction assumption).

### Summary data for exposures

A genome-wide meta-analysis (GWMA) assessed the coffee consumption of up to 91,462 coffee consumers of European ancestry, adjusting for age, smoking status, and, when applicable, sex, case–control status, study site, family structure, and/or study-specific principal components of the population substructure (12). The average daily coffee consumption in this meta-analysis ranged from 1.7 to 5.8 cups.

Another genetic variant dataset associated with coffee consumption was obtained from a genome-wide association study (GWAS) of bitter and sweet beverage consumption among 375,833 participants of European ancestry, adjusting for age, sex, body mass index, total energy, and the top 20 principal components (13). Total daily coffee consumption was obtained by the 24-h dietary recall questionnaire, and the mean intake was calculated based on completing at least two dietary questionnaires.

Because of the availability of caffeine in tea, we additionally obtained genetic variants from a GWAS study, with 407,072 participants’ self-reports on caffeine intake from tea (14). The consumption was calculated by multiplying the number of cups of tea by the caffeine content per cup.

### Summary data for outcome

ED data were derived from a GWAS study conducted by Bovijn et al., which recruited 223,805 European men (6,175 cases and 217,630 controls) by combining three cohorts (15). Another summary association data for ED were obtained from the FinnGen datasets (*N* = 95,178, 1,154 cases and 94,024 controls) (16).

### Selection of instrumental variables

SNPs associated with coffee/caffeine consumption at genome-wide significance (*p*<5×10^−8^) were selected as instrumental variables (IVs). To make SNPs independent of each other, we pruned SNPs by a clumping procedure with *r* ^2^ < 0.001 and kb = 10,000. To avoid violating the independence assumption in MR, PhenoScannerV2 was used to assess whether the retained SNPs were associated with confounding factors such as type 2 diabetes, hypertension, cardiovascular disease, body mass index, and triglycerides (17). *F*-statistics are calculated according to *F* = *R* ^2^ × (*N*−2)/(1−*R* ^2^), and an *F*-statistic of over 10 indicates no weak instrumental bias. The outlier IVs were PRESSO deleted by MR pleiotropy residual sum and outlier (MR-PRESSO) before MR analyses to account for possible pleiotropy (18).

### Statistical analyses

The inverse variance weighted (IVW) method was used as the determinant method of the causal estimate of coffee/caffeine consumption on the risk of ED (19). If significant heterogeneity was detected through Cochran’s *Q* statistic, the random-effect IVW model was adopted; otherwise, the fixed-effect IVW model was adopted (20).

In addition, the other four MR methods, namely, MR-Egger, weighted median, simple mode, and weighted mode (21–23), are powerful supplements to the IVW method, providing more reliable estimates under broader conditions.

MR-Egger regression intercept was performed to detect directional pleiotropy. Leave-one-out analysis was performed to assess whether the casual estimates in the MR analysis were driven by a single SNP, which was achieved by sequentially removing each SNP (24).

Meanwhile, to ensure the robustness of the results, our study was validated in two independent ED datasets, and a meta-analysis was conducted on each exposure factor based on the IVW results from two datasets, using the fixed-effect method.

All MR analyses were performed by using “TwoSampleMR”, “forestploter”, “MR-PRESSO”, “MendelianRandomization”, and “meta” packages in R (version 4.1.3). *p* < 0.05 was considered statistically significant.

## Results

### Characteristics of selected SNPs

By performing a series of selection steps, 3, 22, and 15 SNPs were screened as genetic IVs for assessing the effects of coffee consumption from GWMA, coffee consumption from GWAS, and caffeine from tea on ED (Bovijn datasets), respectively. In addition, we identified 4 SNPs as IVs for coffee consumption from GWMA, 20 SNPs as IVs for coffee consumption from GWAS, and 12 SNPs as IVs for caffeine from tea on ED (FinnGen datasets). Characteristics of SNPs associated with coffee/caffeine consumption and ED are presented in **Supplementary Tables S1** and **S2**.

### Causal effects of coffee/caffeine consumption on the risk of ED

**Figure 2:**
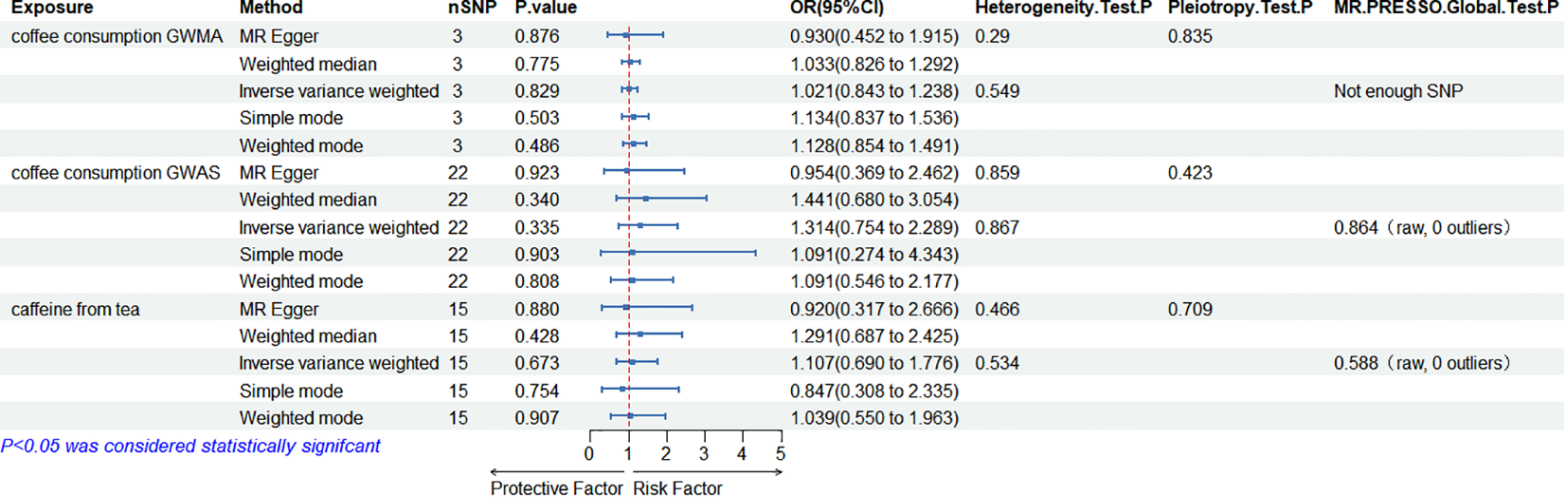
Association of genetically predicted coffee/caffeine consumption with the risk of erectile dysfunction (Bovijn datasets).

When the ED data conducted by the Bovijn study were used as the outcome variable, the MR analysis showed that predisposition to coffee consumption from GWMA (IVW OR=1.021; 95% CI: 0.843–1.238; *p*=0.829), coffee consumption from GWAS (IVW OR=1.314; 95% CI: 0.754–2.289; *p*=0.335), and caffeine from tea (IVW OR=1.107; 95% CI: 0.690–1.776; *p*=0.673) had no causal effect on ED (**Figure 2**; **Table 1**).

**Table 1:** Summary on MR results of coffee/caffeine consumption on erectile dysfunction (Bovijn datasets).

Exposure	Outcome	MR results					Heterogeneity	Pleiotropy	MR-PRESSO
		Methods	No. of SNPs	OR	95%CI	*p*			
Coffee Consumption from GWMA	ED	MR-Egger	3	0.930	0.452–1.915	0.876	0.290	0.835	
		Weighted median	3	1.033	0.826–1.292	0.775			
		IVW	3	1.021	0.843–1.238	0.829	0.549		Not enough SNPs
		Simple mode	3	1.134	0.837–1.536	0.503			
		Weighted mode	3	1.128	0.854–1.491	0.486			
Coffee Consumption from GWAS	ED	MR-Egger	22	0.954	0.369–2.462	0.923	0.859	0.423	
		Weighted median	22	1.441	0.680–3.054	0.340			
		IVW	22	1.314	0.754–2.289	0.335	0.867		0.864 (raw, 0 outliers)
		Simple mode	22	1.091	0.274–4.434	0.903			
		Weighted mode	22	1.091	0.546–2.177	0.808			
Caffeine from Tea	ED	MR-Egger	15	0.920	0.317–2.666	0.880	0.466	0.709	
		Weighted median	15	1.291	0.687–2.425	0.428			
		IVW	15	1.107	0.690–1.776	0.673	0.534		0.588 (raw, 0 outliers)
		Simple mode	15	0.847	0.308–2.335	0.754			
		Weighted mode	15	1.039	0.550–1.963	0.907			

MR, Mendelian randomization; SNP, single-nucleotide polymorphism; OR, odds ratio; IVW, inverse variance weighted; ED, erectile dysfunction.

**Figure 3:**
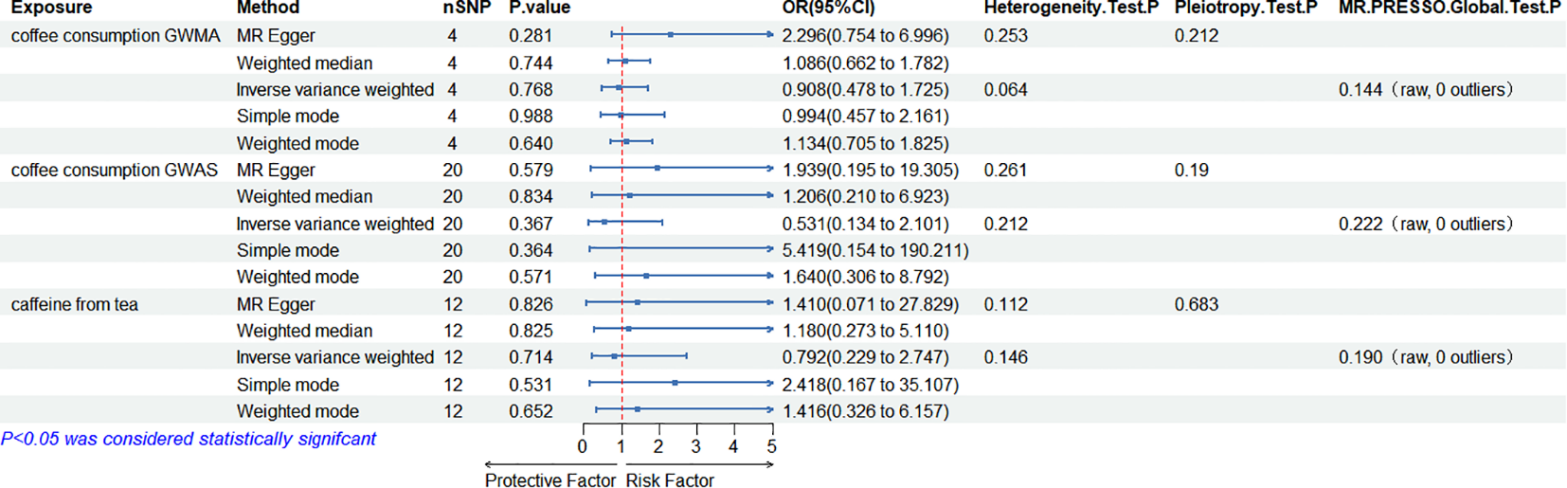
Association of genetically predicted coffee/caffeine consumption with the risk of erectile dysfunction (FinnGen datasets).

When the ED data from FinnGen datasets were used as the outcome variable, the MR analysis showed that predisposition to coffee consumption from GWMA (IVW OR=0.908; 95% CI: 0.478–1.725; *p*=0.768), coffee consumption from GWAS (IVW OR=0.531; 95% CI: 0.134–2.101; *p*=0.367), and caffeine from tea (IVW OR=0.792; 95% CI: 0.229–2.747; *p*=0.531) had no causal effect on ED (**Figure 3**; **Table 2**).

**Table 2:** Summary on MR results of coffee/caffeine consumption on erectile dysfunction (FinnGen datasets).

Exposure	Outcome	MR results					Heterogeneity	Pleiotropy	MR-PRESSO
		Methods	No. of SNPs	OR	95%CI	*p*			
Coffee Consumption from GWMA	ED	MR-Egger	4	2.296	0.754–6.996	0.281	0.253	0.212	
		Weighted median	4	1.086	0.662–1.782	0.744			
		IVW	4	0.908	0.478–1.725	0.768	0.064		0.144 (raw, 0 outliers)
		Simple mode	4	0.994	0.457–2.161	0.988			
		Weighted mode	4	1.134	0.705–1.825	0.640			
Coffee Consumption from GWAS	ED	MR-Egger	20	1.939	0.195–19.305	0.579	0.261	0.190	
		Weighted median	20	1.206	0.210–6.923	0.834			
		IVW	20	0.531	0.134–2.101	0.367	0.212		0.222 (raw, 0 outliers)
		Simple mode	20	5.419	0.154–190.211	0.364			
		Weighted mode	20	1.640	0.306–8.792	0.571			
Caffeine from Tea	ED	MR-Egger	12	1.410	0.071–27.829	0.826	0.112	0.683	
		Weighted median	12	1.180	0.273–5.110	0.825			
		IVW	12	0.792	0.229–2.747	0.714	0.146		0.190 (raw, 0 outliers)
		Simple mode	12	2.418	0.167–35.107	0.531			
		Weighted mode	12	1.416	0.326–6.157	0.652			

MR, Mendelian randomization; SNP, single-nucleotide polymorphism; OR, odds ratio; IVW, inverse variance weighted; ED, erectile dysfunction.

The relevant figures of genetic association between coffee/caffeine consumption and ED can be found in the Supplementary Materials (**Supplementary Figures S1**-**S24**).

### Sensitivity analysis

Cochran’s *Q* statistics showed that the *p*-values of those outcomes were over 0.05, indicating no heterogeneity in IVs. No outlier IVs were detected in our MR analysis, and MR-Egger regression analysis showed no obvious evidence of directional pleiotropy (**Tables 1**, **2**). Additionally, leave-one-out analysis did not detect the casual estimates in the MR analysis that were driven by a single SNP.

### Meta-analysis

**Figure 4:**
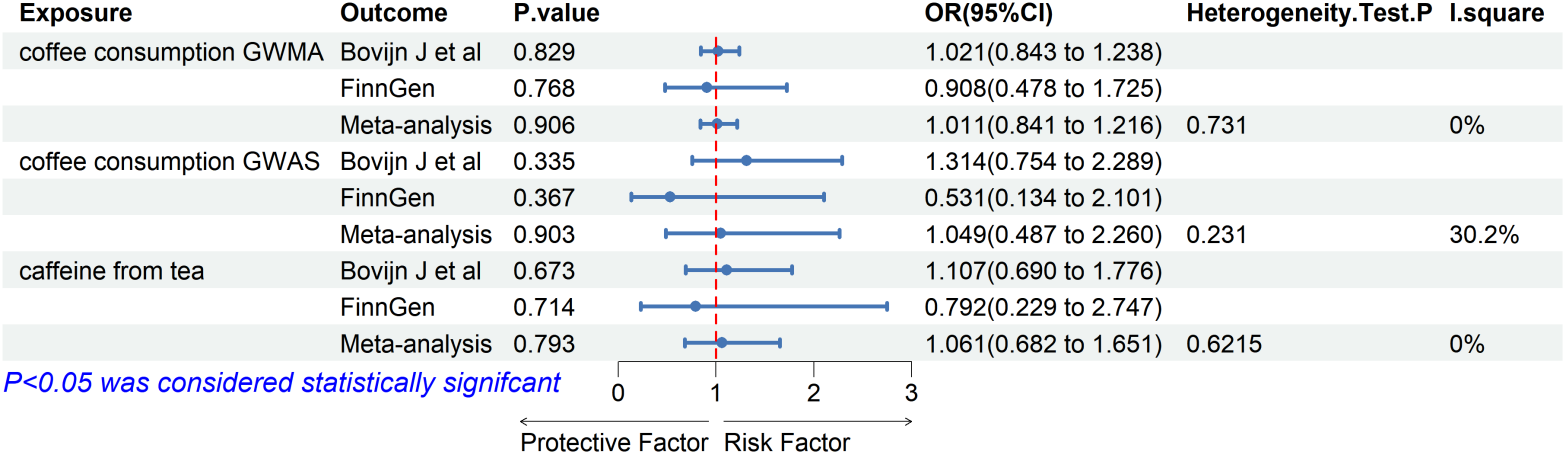
Association of genetically predicted coffee/caffeine consumption with risk of erectile dysfunction. Estimates were obtained from the inverse variance weighted methods.

Based on the two ED datasets, we performed a meta-analysis of the IVW results obtained from the MR analysis and did not detect a causal relationship between coffee/caffeine consumption and ED (coffee consumption from GWMA: OR: 1.011, 95% CI 0.841–1.216, *p*=0.906; coffee consumption from GWAS: OR: 1.049, 95% CI 0.487–2.260, *p*=0.903; caffeine from tea: OR: 1.061, 95% CI 0.682–1.651, *p*=0.793), further indicating that coffee/caffeine consumption was not an important contributor to the occurrence of ED (**Figure 4**).

## Discussion

As reported in epidemiological studies, ED is highly prevalent in men, and its incidence increases with age (3). This personal disease often causes anxiety and potential depression in patients with ED, which brings significant psychological and economic pressure, and has become a substantial health problem (25). Therefore, it is critical to identify the risk factors and susceptible populations associated with ED.

In a combined sample of two ED datasets, the results of our analysis did not support the causality between genetically predicted coffee/caffeine consumption and the risk of ED, which was consistent with the findings of most studies (9, 26, 27). A large-scale study using the Health Professionals Follow-up Study from 1998 to 2010, did not find an association between total coffee consumption and ED, with similar associations for regular coffee consumption (27). In a multivariate analysis including 350 individuals, after adjusting for age, body mass index, and smoking status, coffee consumption was not associated with the risk of incident ED (9). No association between coffee consumption and ED was found in a prospective study of 202 patients with 5 years of follow-up (26). Furthermore, another MR study, using a single caffeine dataset, found no causal relationship between caffeine and ED (28).

However, a case–control study including 3,724 participants found a negative association between caffeine intake and the incidence of ED, particularly at levels corresponding to about two to three cups of coffee per day (6). A population-based study from Turkey also found a negative association between caffeine intake and the risk of ED (29). Compared to these studies, our research includes a larger sample size of ED patients. By using the MR method, we minimized bias due to residual confounding and avoided reverse causation inherent in observational designs. Furthermore, compared to the study by Lopez et al., our research incorporated additional confounding factors, such as cardiovascular disease. Finally, we conducted a meta-analysis based on MR, which is more robust than a single study and provides more reliable results.

Although the association between caffeine consumption and ED is still unclear, several pharmacological effects of caffeine are thought to be involved in regulating ED. Caffeine is a nonselective phosphodiesterase inhibitor commonly found in coffee, which could elevate the level of intracellular cyclic guanosine monophosphate (30, 31). Caffeine could relax cavernous smooth muscles and decrease the contractile effects of cavernous muscle strips, due to the reduction of calcium ion flow (32). In addition, coffee, potentially due to its abundant antioxidants, alleviates the toxic effects of oxidative stress and inflammation within diverse tissues via the activation of nuclear factor erythroid 2-related factor-2, to enhance its potential benefits, especially in the field of cardiovascular disease (33). In a sense, coffee might also have potential benefits on ED as well as protective effects on cardiovascular disease.

It is well known that testosterone plays a crucial role in male erectile function. A previous finding has also shown that caffeine has the potential to increase testosterone levels (34). The study observed a more pronounced increase in testosterone levels following caffeine intake, suggesting that adenosine may play a mediating role in the performance-enhancing effects of caffeine. Interestingly, a cross-sectional study with a nationally representative sample of adult men in the United States observed a negative correlation between caffeine and serum testosterone (35). However, the specific sources of caffeine in this population (such as coffee, tea, or soda) could not be determined. Future research is warranted to further validate these findings.

There are some strengths in our study. Firstly, the major advantage is the MR design, employing IVs to explore the causality of exposure on the outcome, which reduced the potential confounding and reverse causation bias and thus strengthened the causal inference in the associations between coffee/caffeine consumption with risk of ED. Secondly, we examined these associations in two datasets to ensure a substantial sample size, and further ensured the robustness of findings through a meta-analysis. Thirdly, potential bias was reduced by using sensitivity analyses.

However, our analysis does have some limitations. Firstly, exposure and outcome data are derived from European populations, and hence, this finding could not be generalized to the general population. Secondly, heterogeneity and pleiotropy are two major concerns in MR analysis. Although we conducted rigorous instrument selection, utilized multiple analytical methods, and performed multiple sensitivity analyses, the potential for bias remains. Therefore, the results should be interpreted with caution. Third, although we used the only two independent ED GWAS datasets, the relatively low ED case rate in these datasets may have led to reduced statistical power. This limitation can be somewhat alleviated by conducting a meta-analysis of these results. Therefore, further validation with a larger sample size should be considered.

## Conclusion

In this MR study, we found no strong evidence to support the associations between coffee/caffeine consumption and the risk of ED in the European population. However, genetic or epidemiological mechanisms underlying their relationships remain unknown, and further studies are warranted to validate our MR findings and investigate the underlying mechanisms.

## Data Availability

The original contributions presented in the study are included in the article/**Supplementary Material**. Further inquiries can be directed to the corresponding author.

## Supplementary material

Supplementary Figure 1–4 The plots of the effect of coffee consumption GWMA on erectile dysfunction (Bovijn datasets).

Supplementary Figure 5–8 The plots of the effect of coffee consumption GWAS on erectile dysfunction (Bovijn datasets).

Supplementary Figure 9–12 The plots of the effect of caffeine from tea on erectile dysfunction (Bovijn datasets).

Supplementary Figure 13–16 The plots of the effect of coffee consumption GWMA on erectile dysfunction (FinnGen datasets).

Supplementary Figure 17–20 The plots of the effect of coffee consumption GWAS on erectile dysfunction (FinnGen datasets).

Supplementary Figure 21–24 The plots of the effect of caffeine from tea on erectile dysfunction (FinnGen datasets).
