# Supplementary figures and images for: Potential genetic association between coffee/caffeine consumption and erectile dysfunction: a Mendelian randomization study and meta-analysis
Source: Front Endocrinol (Lausanne). 2024 Oct 9;15:1400491. doi: 10.3389/fendo.2024.1400491 (PMC11497819; doi:10.3389/fendo.2024.1400491)

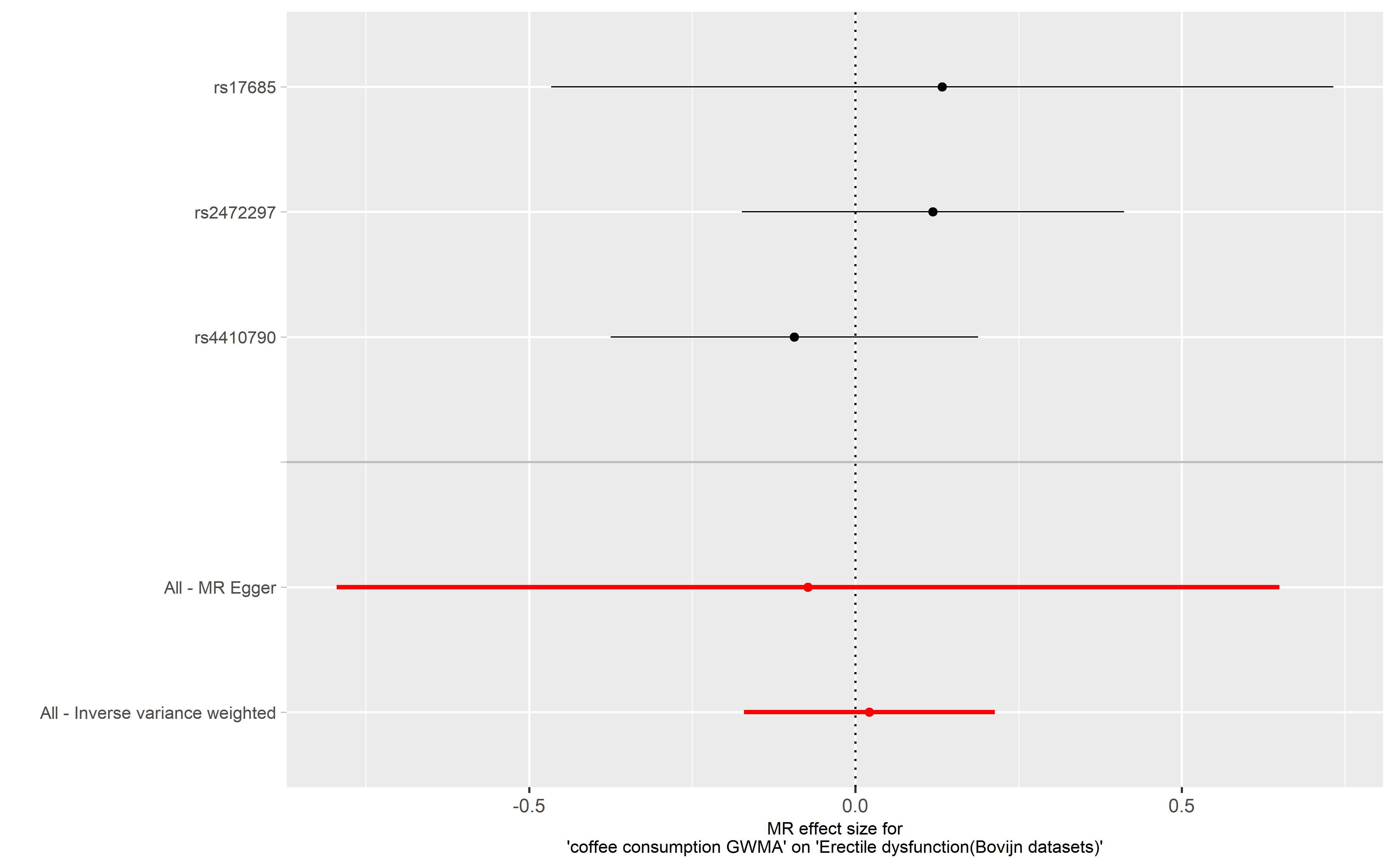

Supplement: Supplementary Figure 1–4 — The plots of the effect of coffee consumption GWMA on erectile dysfunction (Bovijn datasets). [file DataSheet1.zip › Supplementary information/Supplementary Figure/Figure S1.tif]

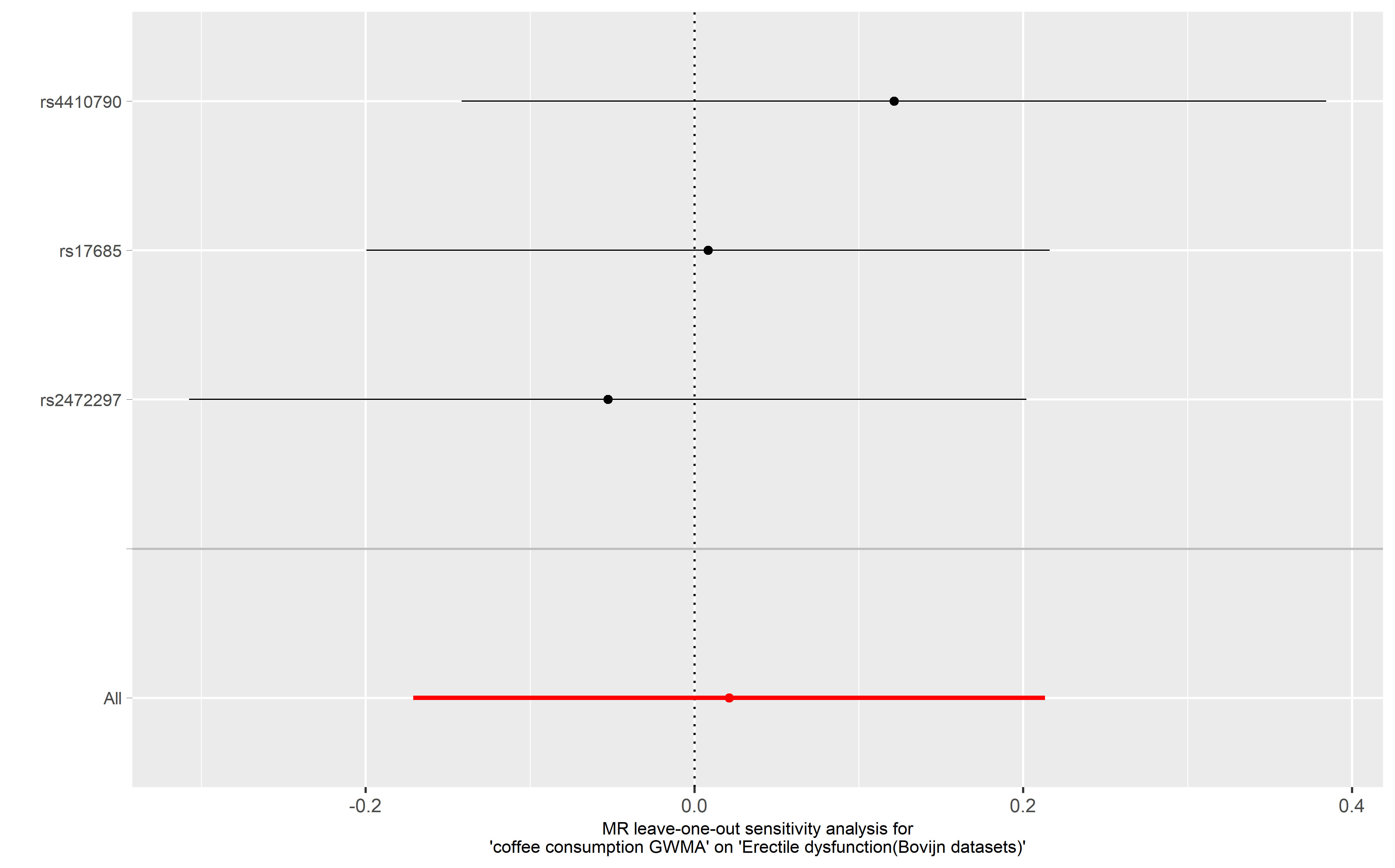

Supplement: Supplementary Figure 1–4 — The plots of the effect of coffee consumption GWMA on erectile dysfunction (Bovijn datasets). [file DataSheet1.zip › Supplementary information/Supplementary Figure/Figure S2.tif]

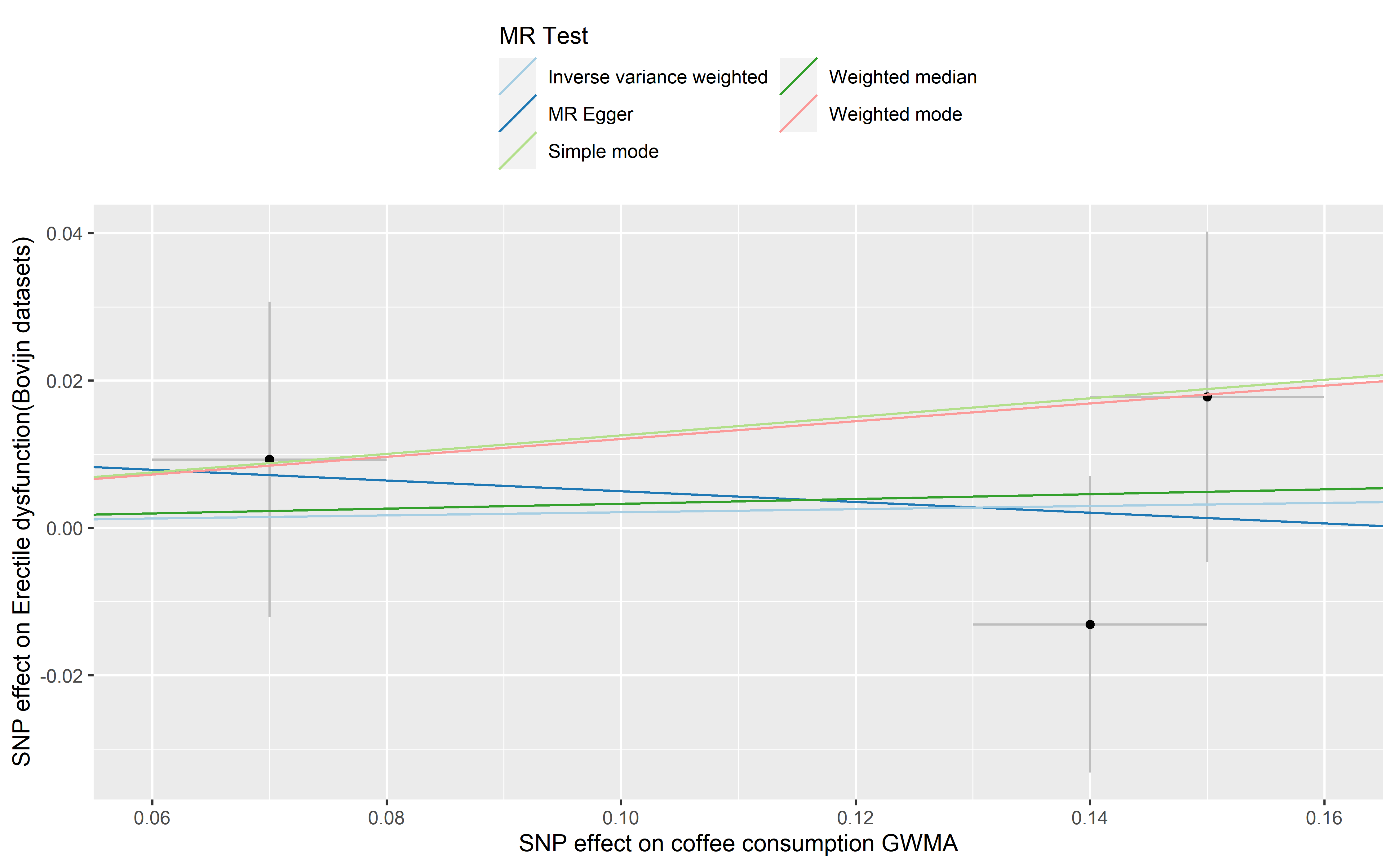

Supplement: Supplementary Figure 1–4 — The plots of the effect of coffee consumption GWMA on erectile dysfunction (Bovijn datasets). [file DataSheet1.zip › Supplementary information/Supplementary Figure/Figure S3.tif]

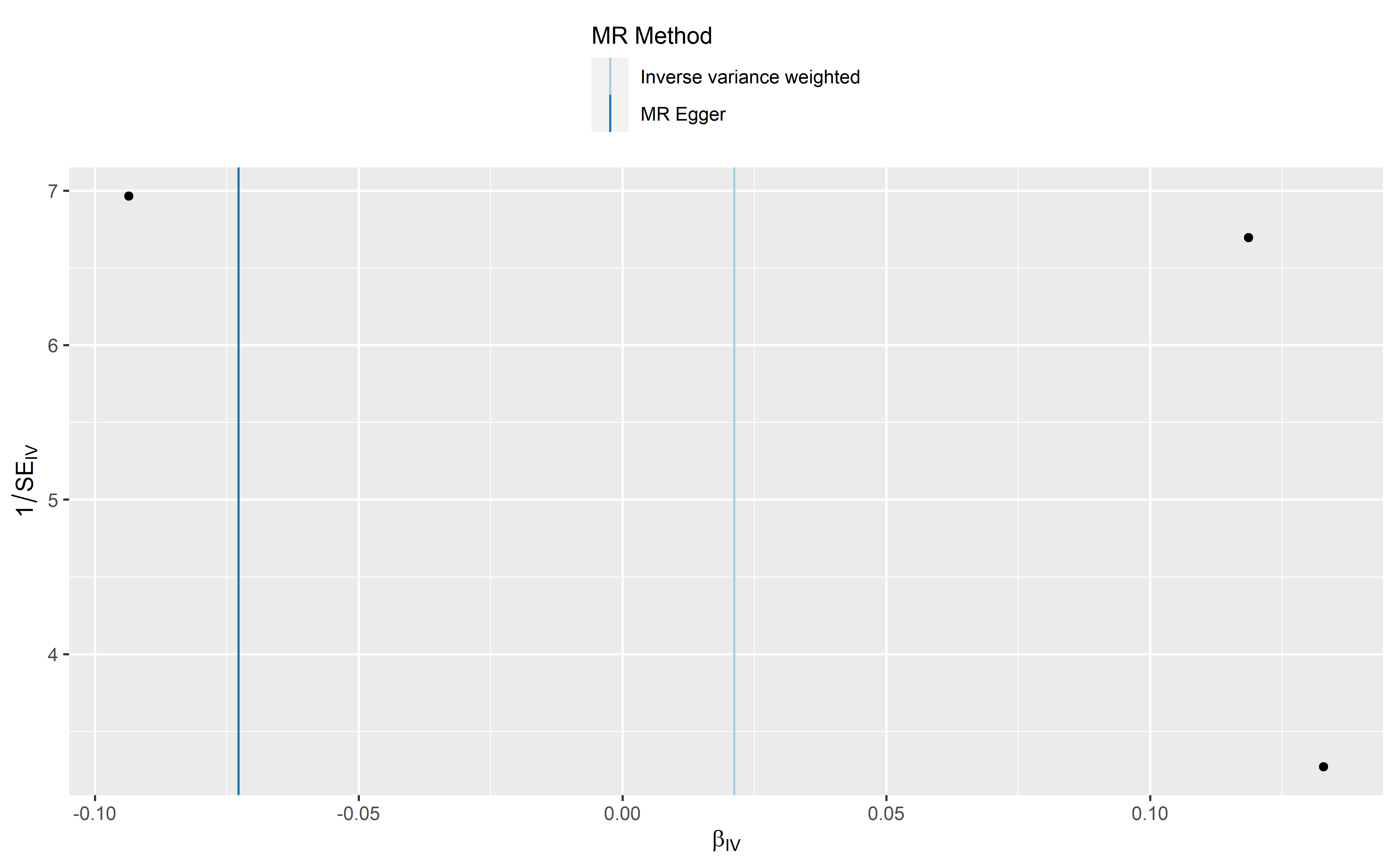

Supplement: Supplementary Figure 1–4 — The plots of the effect of coffee consumption GWMA on erectile dysfunction (Bovijn datasets). [file DataSheet1.zip › Supplementary information/Supplementary Figure/Figure S4.tif]

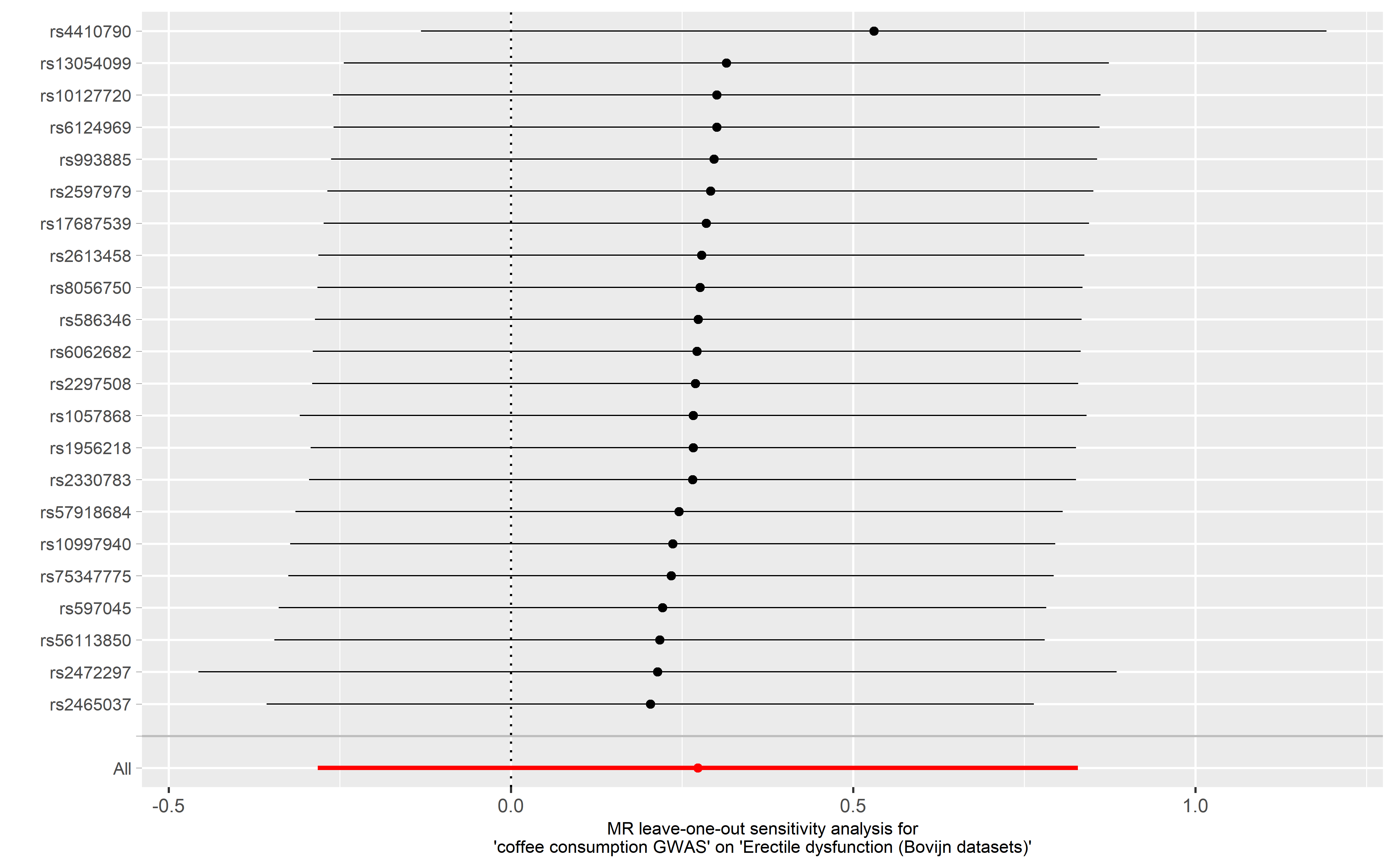

Supplement: Supplementary Figure 1–4 — The plots of the effect of coffee consumption GWMA on erectile dysfunction (Bovijn datasets). [file DataSheet1.zip › Supplementary information/Supplementary Figure/FigureS10.tif]

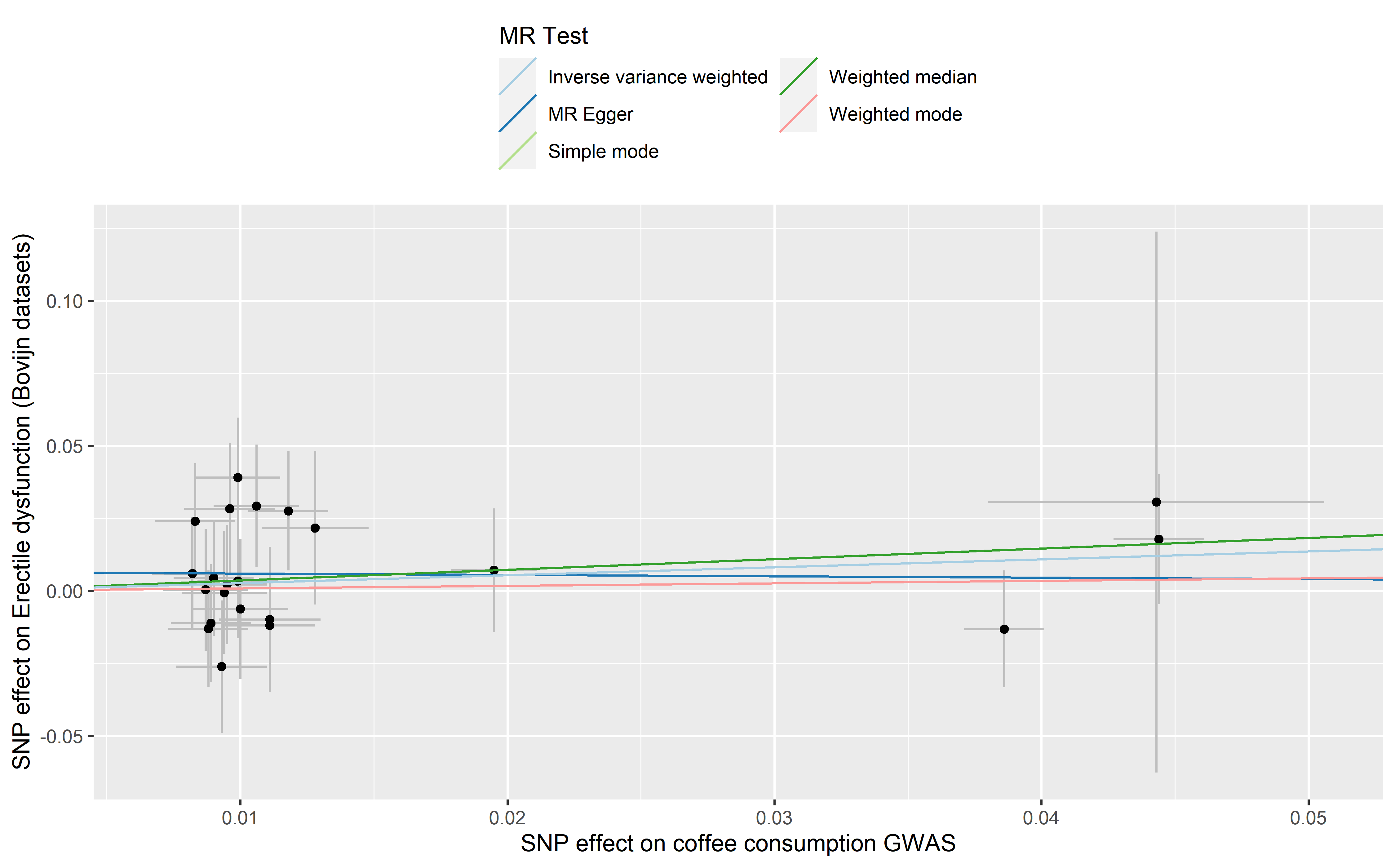

Supplement: Supplementary Figure 1–4 — The plots of the effect of coffee consumption GWMA on erectile dysfunction (Bovijn datasets). [file DataSheet1.zip › Supplementary information/Supplementary Figure/FigureS11.tif]

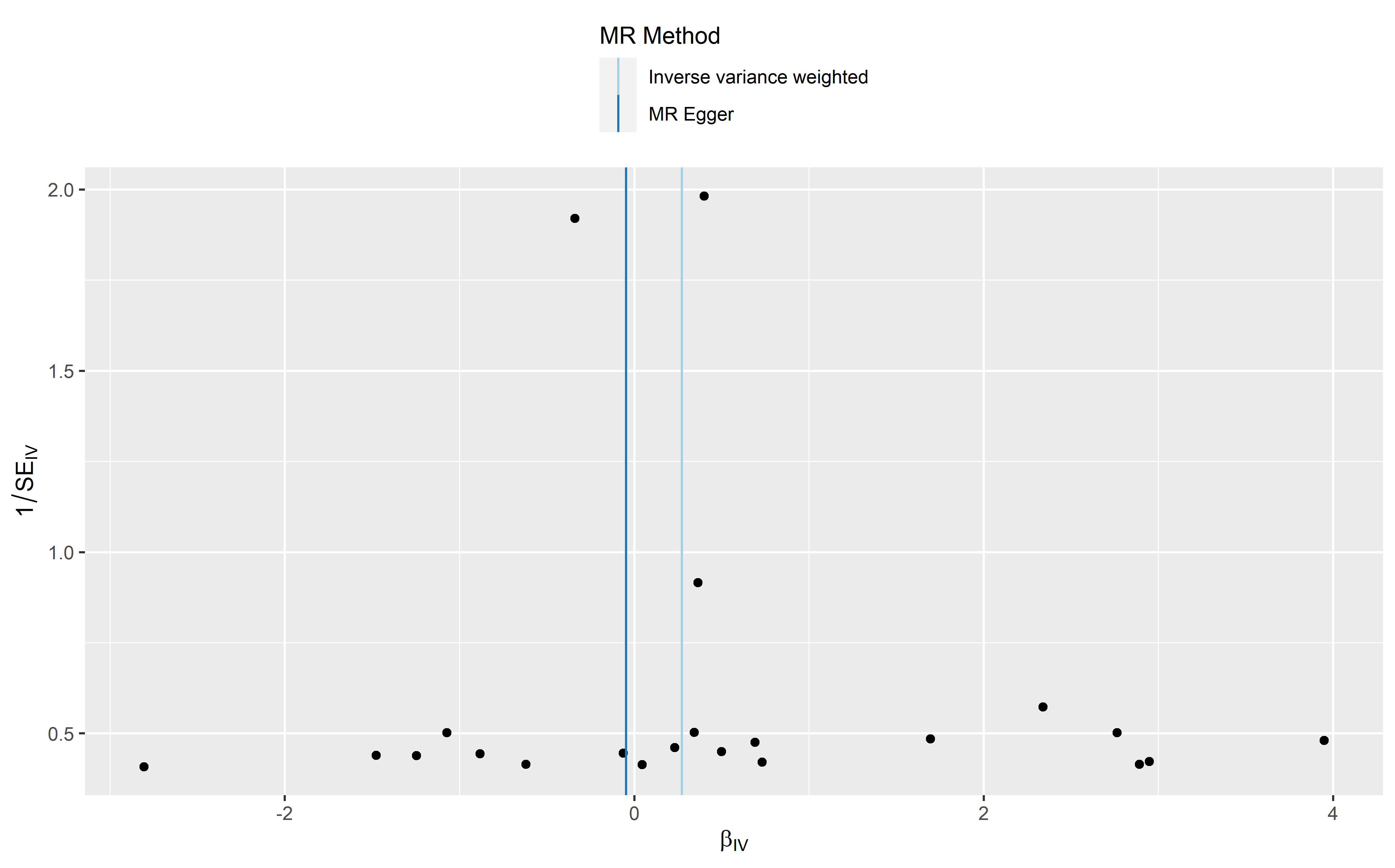

Supplement: Supplementary Figure 1–4 — The plots of the effect of coffee consumption GWMA on erectile dysfunction (Bovijn datasets). [file DataSheet1.zip › Supplementary information/Supplementary Figure/FigureS12.tif]

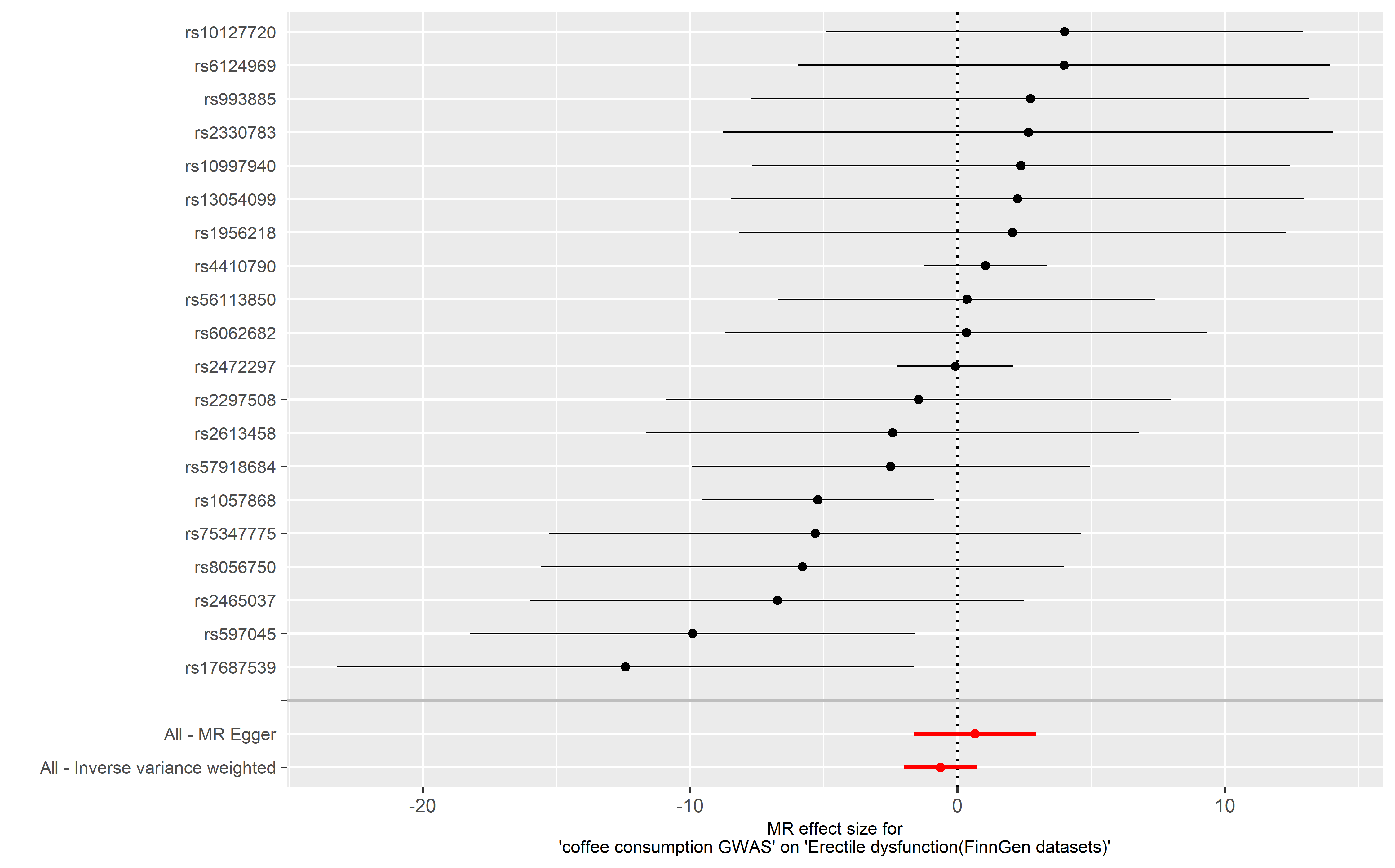

Supplement: Supplementary Figure 1–4 — The plots of the effect of coffee consumption GWMA on erectile dysfunction (Bovijn datasets). [file DataSheet1.zip › Supplementary information/Supplementary Figure/FigureS13.tif]

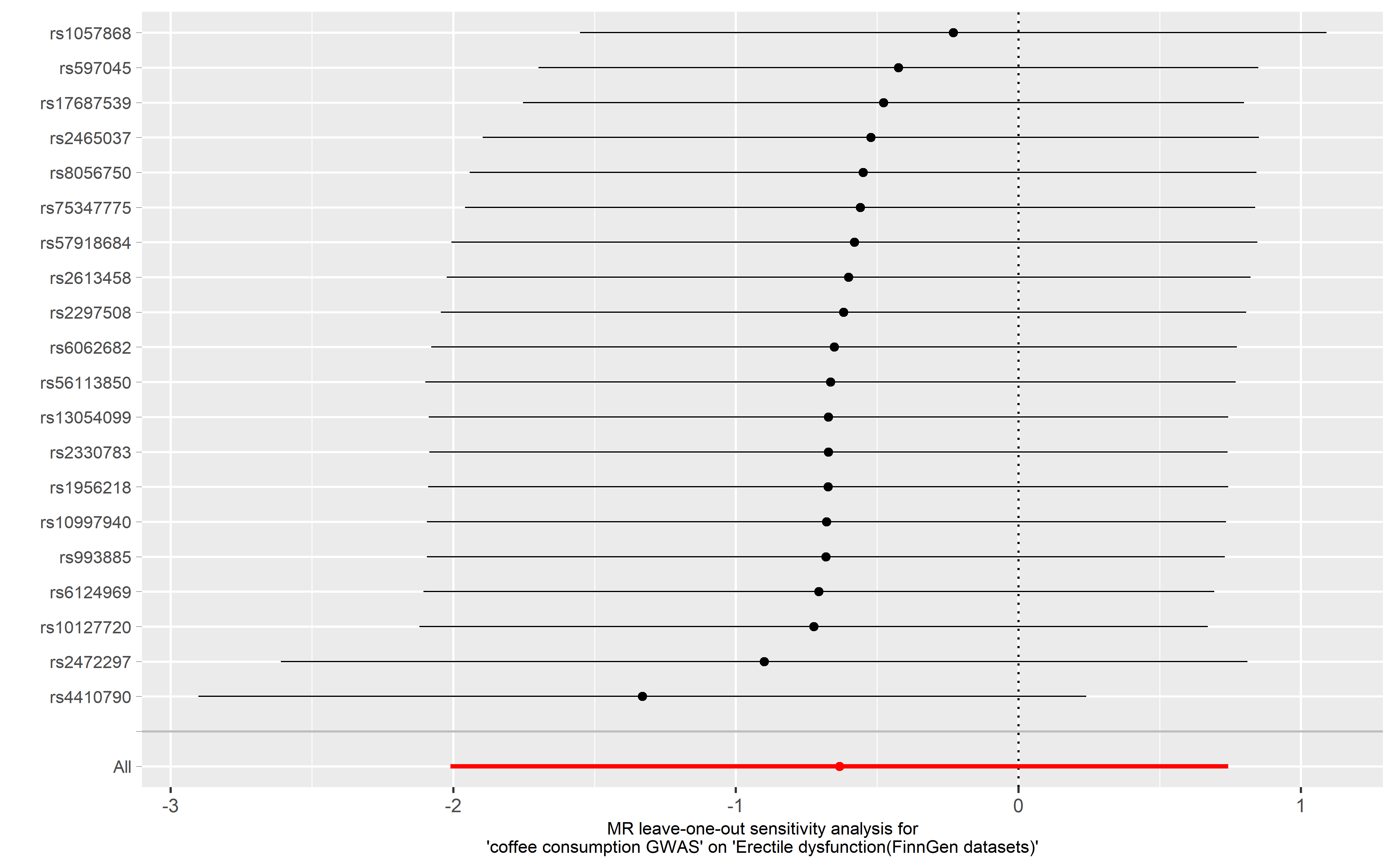

Supplement: Supplementary Figure 1–4 — The plots of the effect of coffee consumption GWMA on erectile dysfunction (Bovijn datasets). [file DataSheet1.zip › Supplementary information/Supplementary Figure/FigureS14.tif]

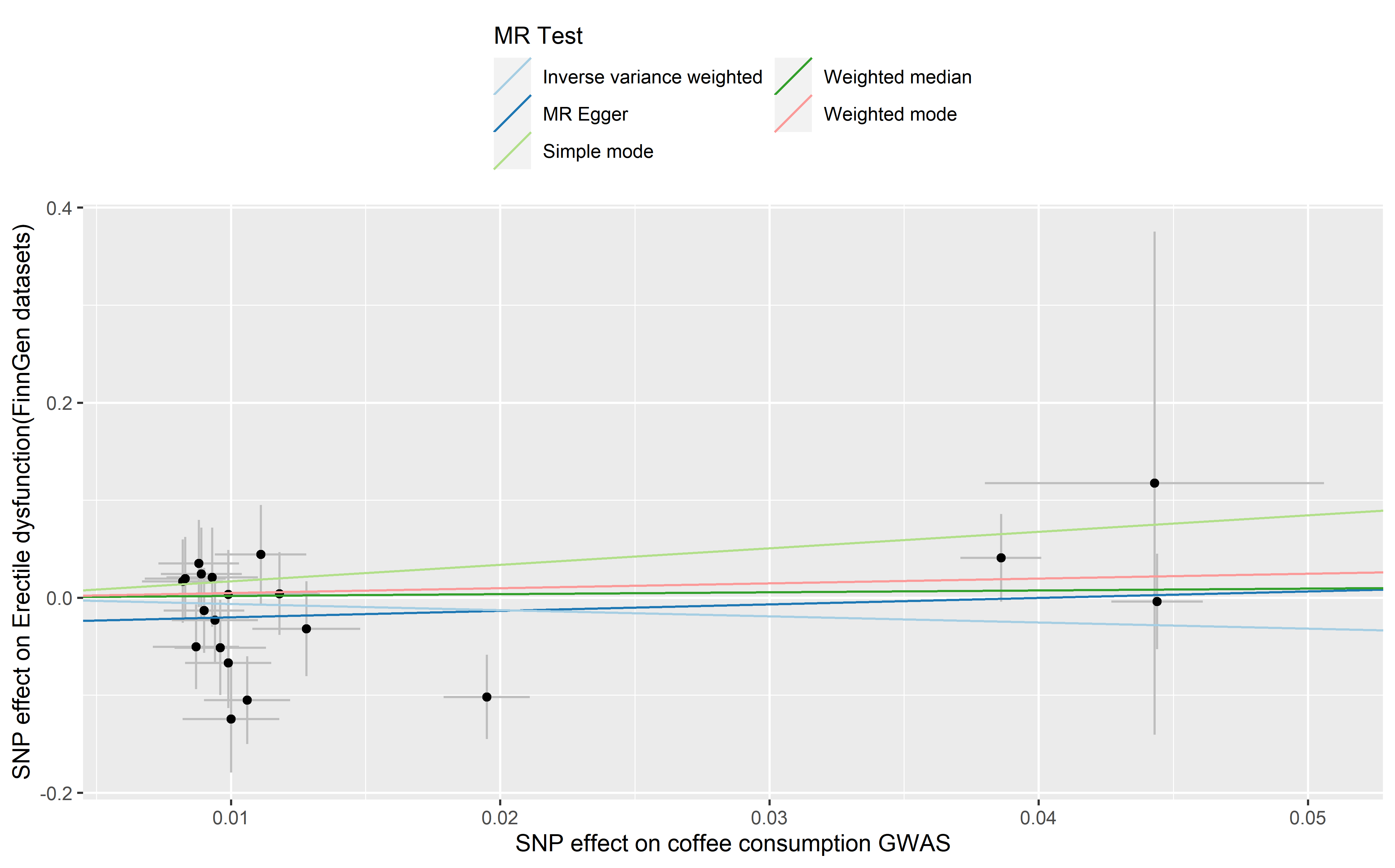

Supplement: Supplementary Figure 1–4 — The plots of the effect of coffee consumption GWMA on erectile dysfunction (Bovijn datasets). [file DataSheet1.zip › Supplementary information/Supplementary Figure/FigureS15.tif]

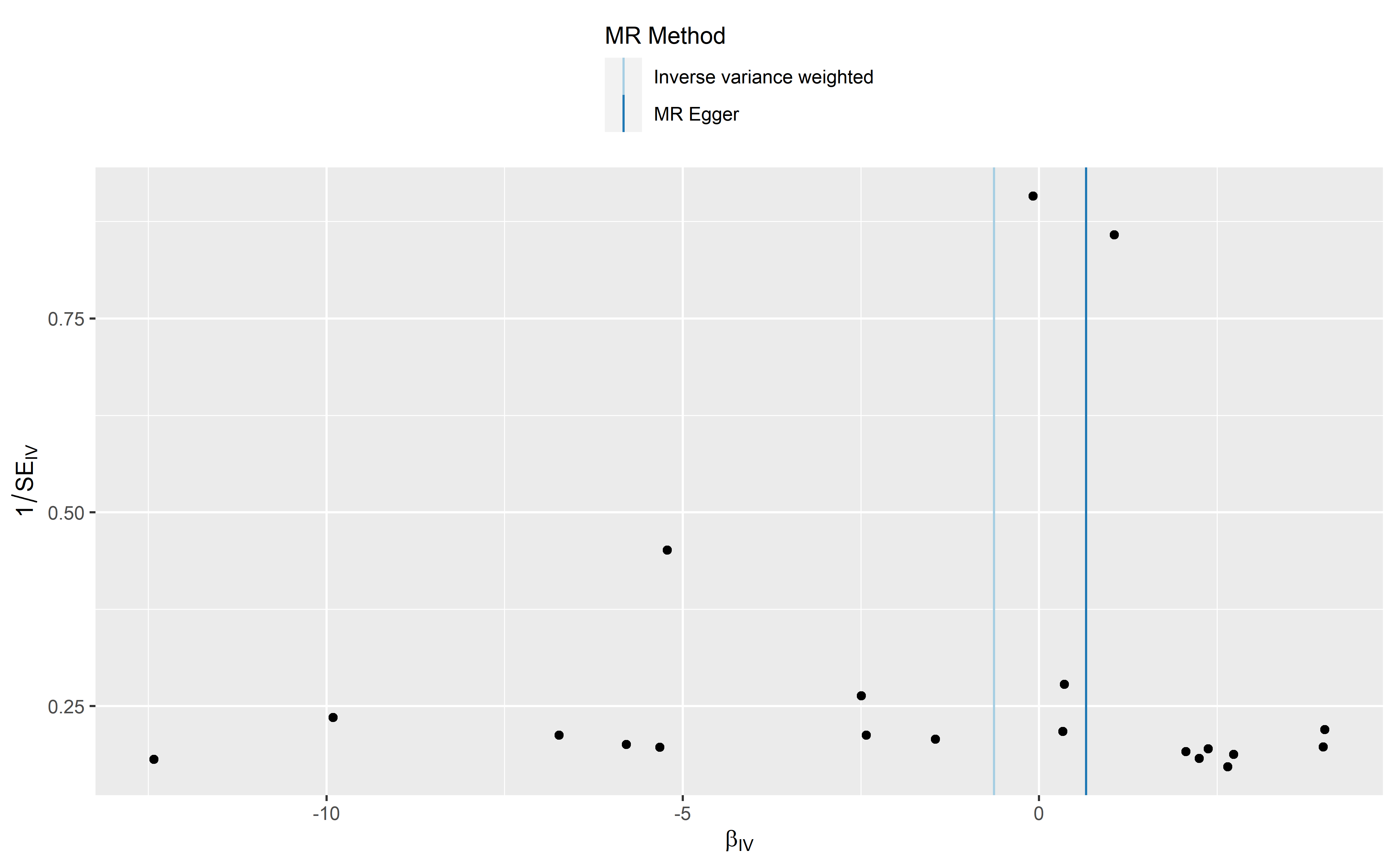

Supplement: Supplementary Figure 1–4 — The plots of the effect of coffee consumption GWMA on erectile dysfunction (Bovijn datasets). [file DataSheet1.zip › Supplementary information/Supplementary Figure/FigureS16.tif]

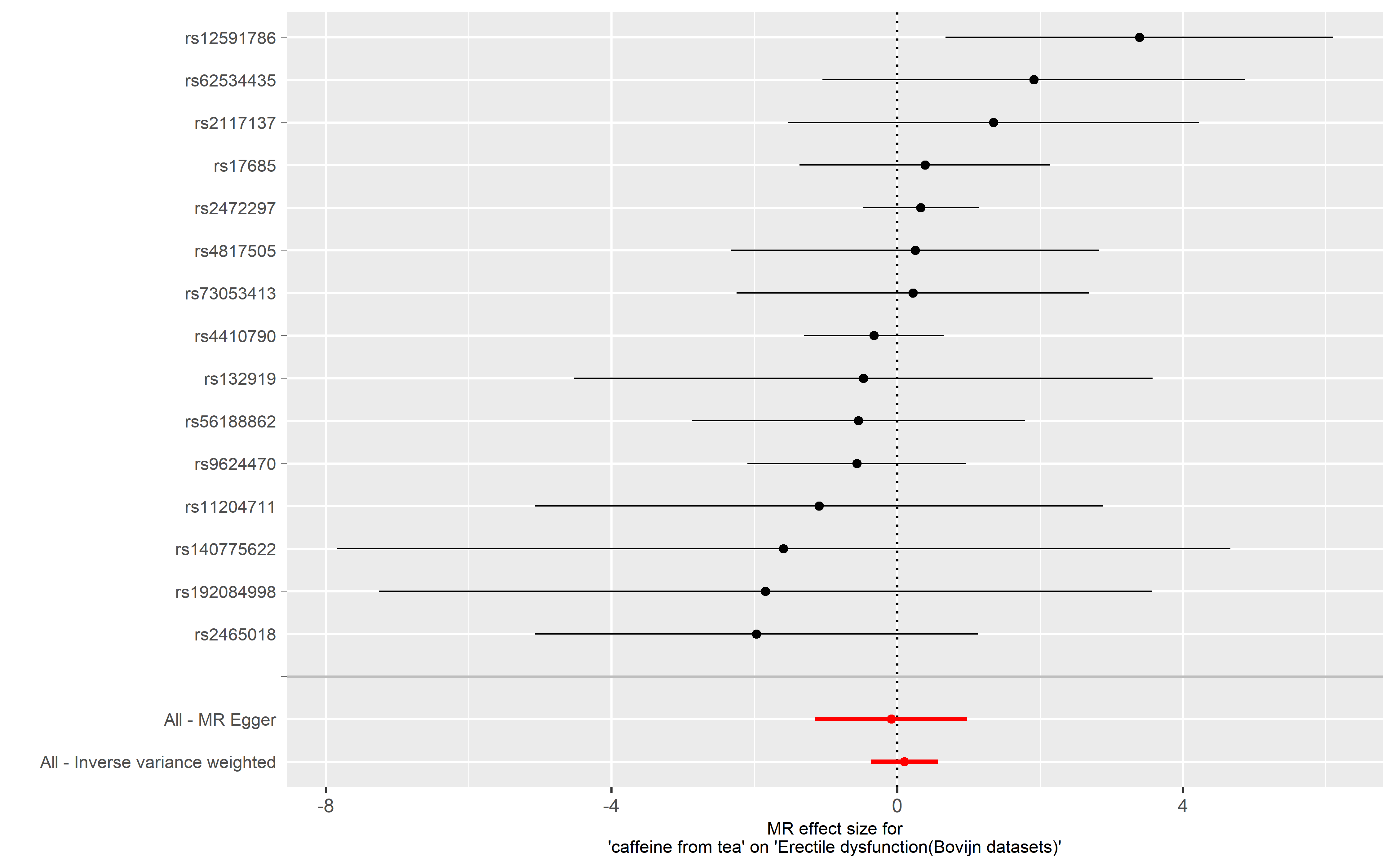

Supplement: Supplementary Figure 1–4 — The plots of the effect of coffee consumption GWMA on erectile dysfunction (Bovijn datasets). [file DataSheet1.zip › Supplementary information/Supplementary Figure/FigureS17.tif]

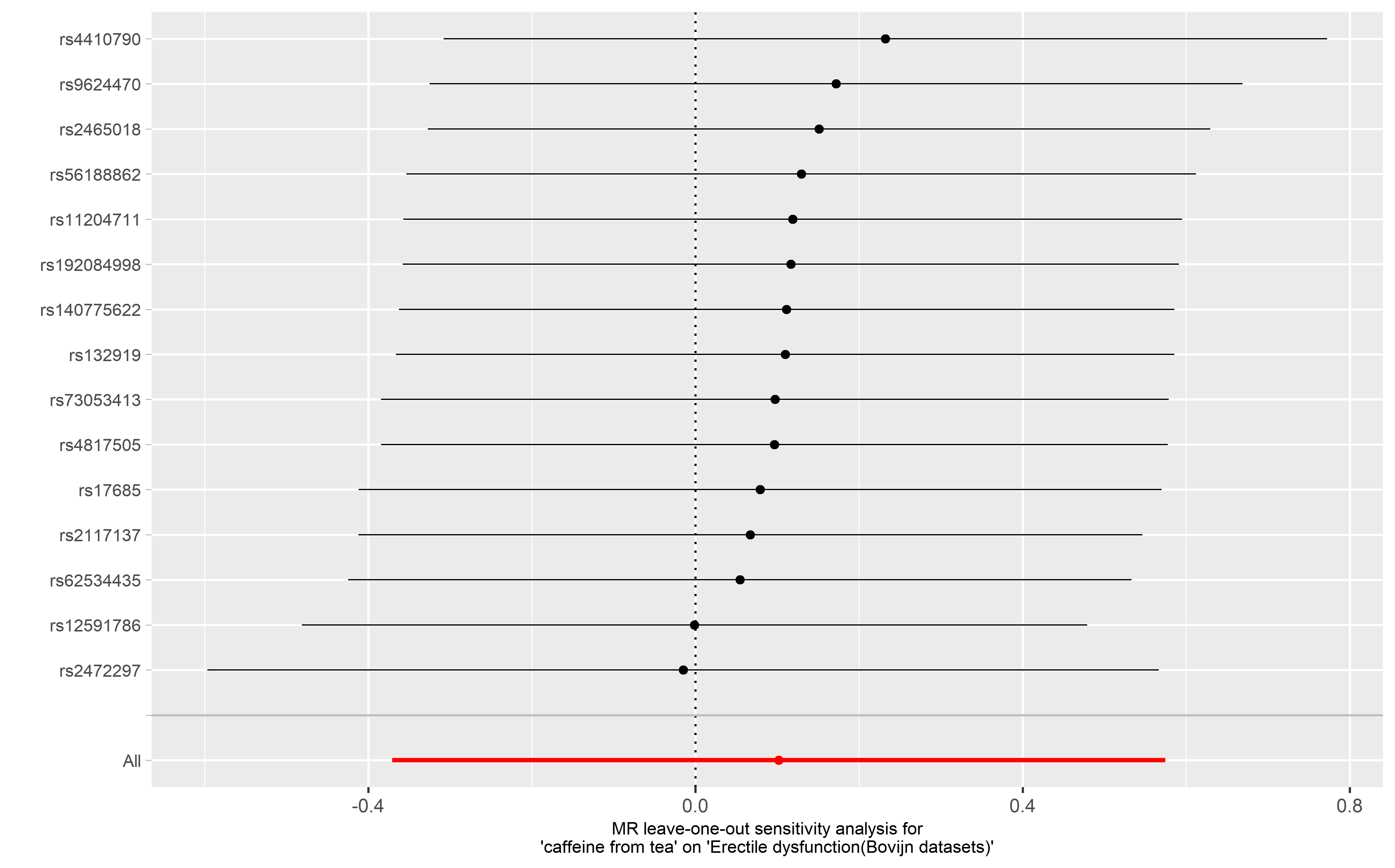

Supplement: Supplementary Figure 1–4 — The plots of the effect of coffee consumption GWMA on erectile dysfunction (Bovijn datasets). [file DataSheet1.zip › Supplementary information/Supplementary Figure/FigureS18.tif]

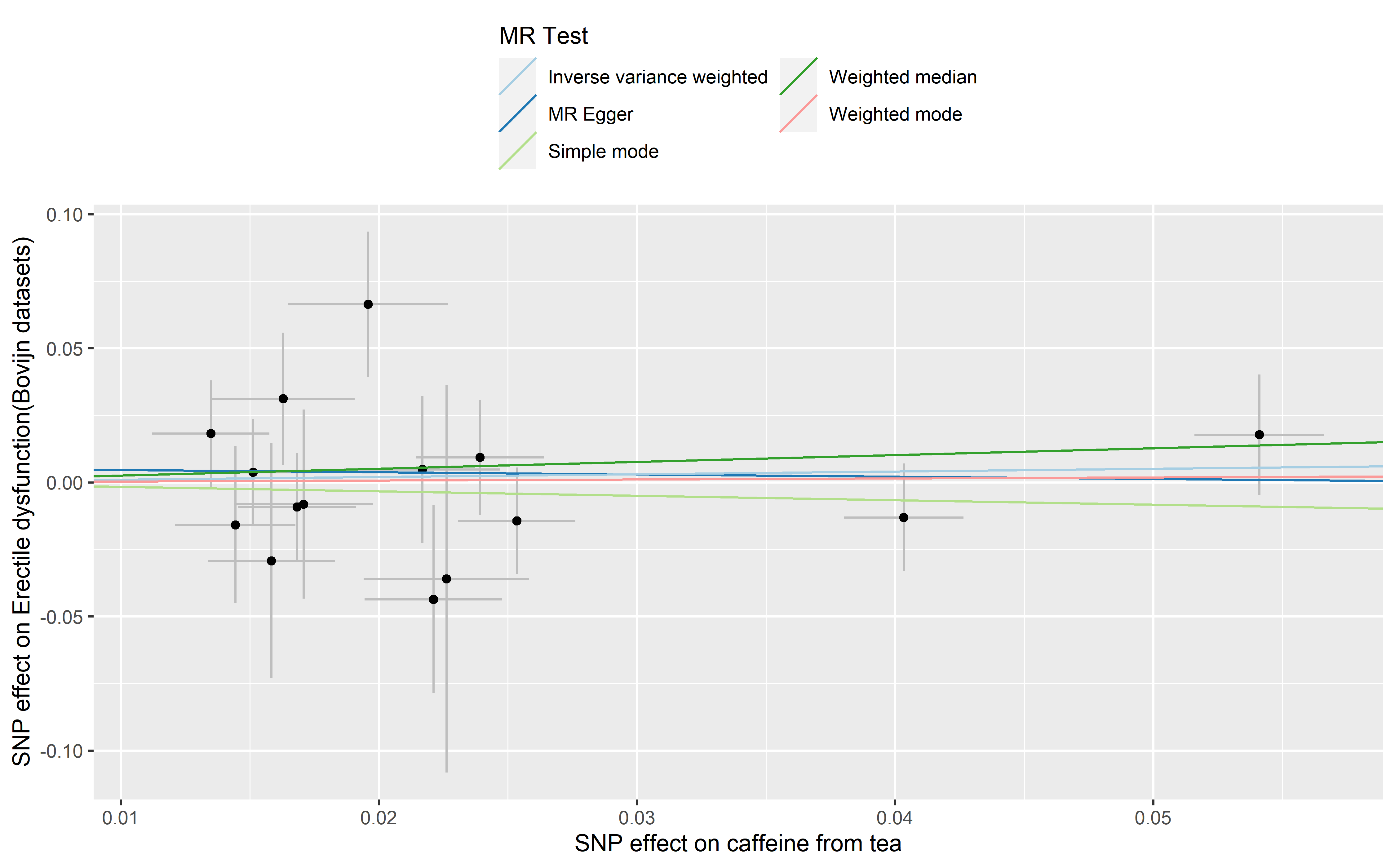

Supplement: Supplementary Figure 1–4 — The plots of the effect of coffee consumption GWMA on erectile dysfunction (Bovijn datasets). [file DataSheet1.zip › Supplementary information/Supplementary Figure/FigureS19.tif]

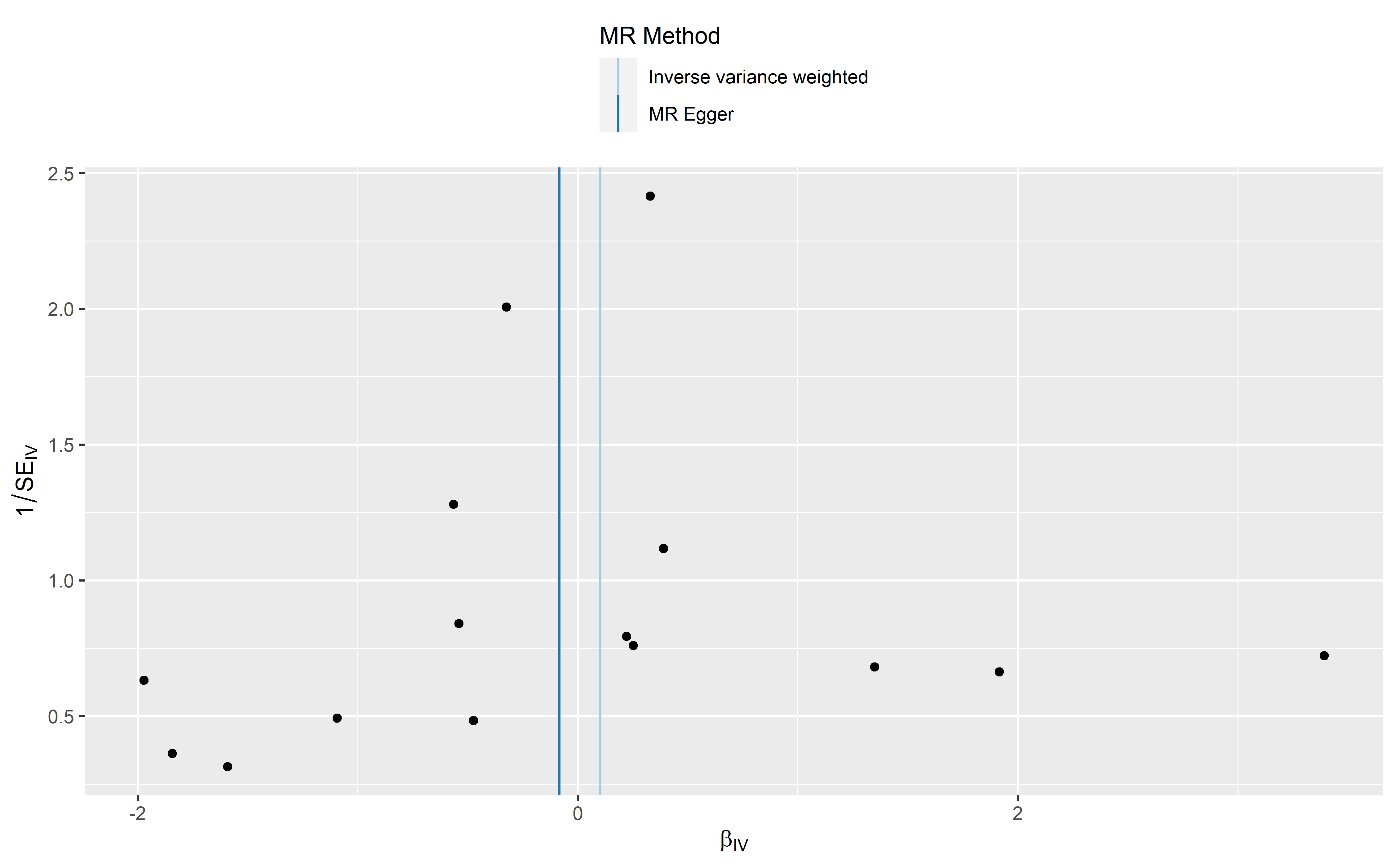

Supplement: Supplementary Figure 1–4 — The plots of the effect of coffee consumption GWMA on erectile dysfunction (Bovijn datasets). [file DataSheet1.zip › Supplementary information/Supplementary Figure/FigureS20.tif]

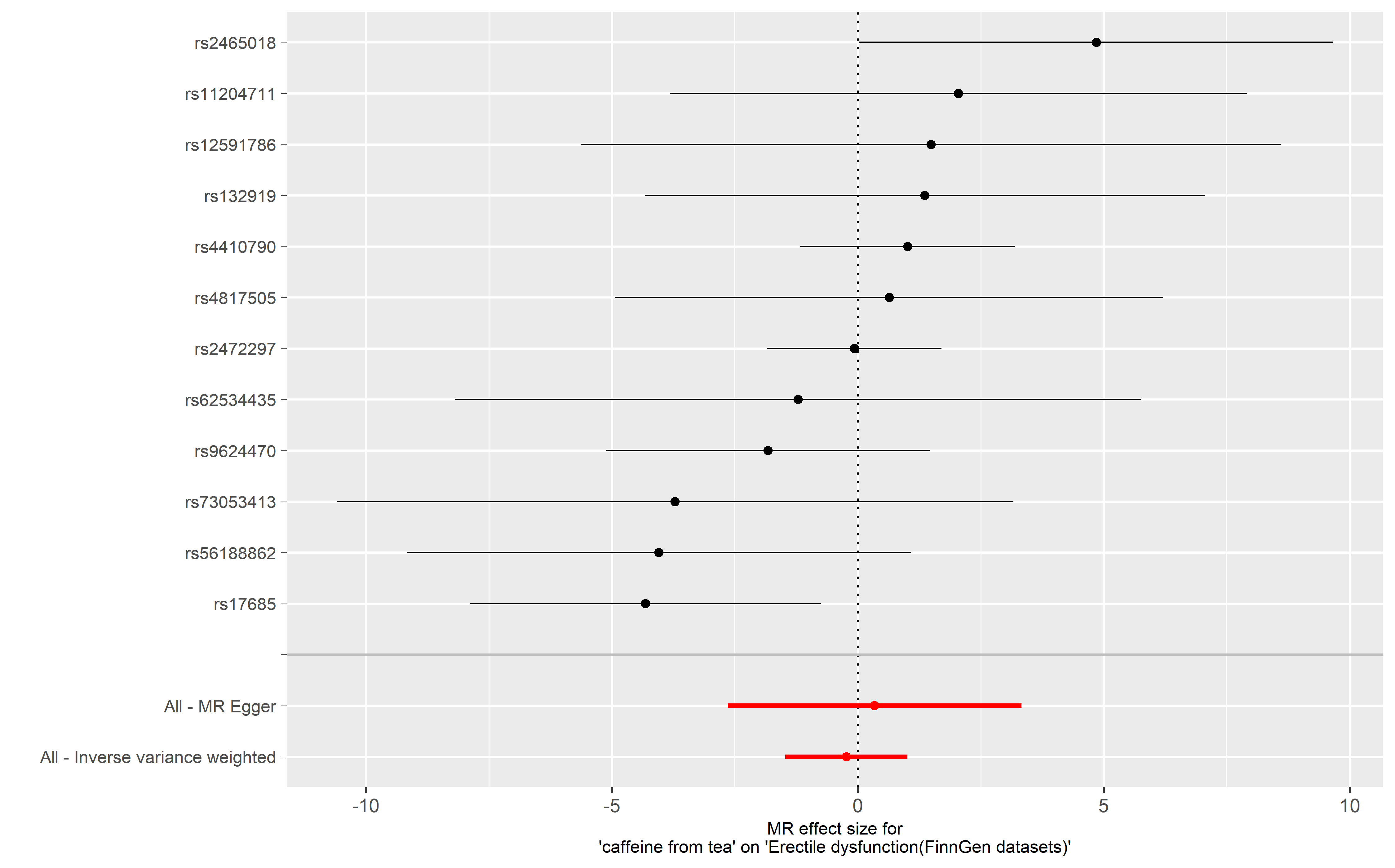

Supplement: Supplementary Figure 1–4 — The plots of the effect of coffee consumption GWMA on erectile dysfunction (Bovijn datasets). [file DataSheet1.zip › Supplementary information/Supplementary Figure/FigureS21.tif]

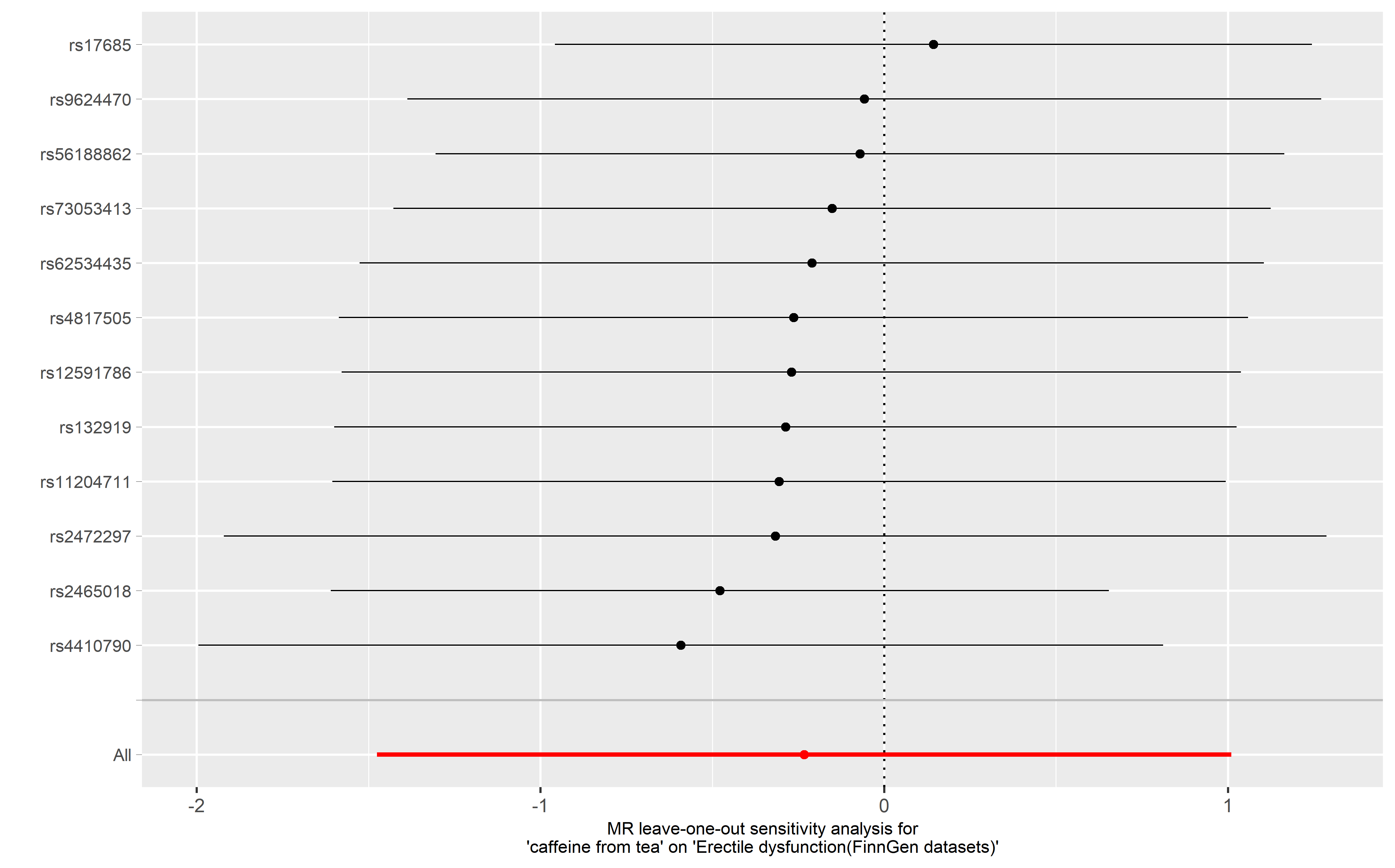

Supplement: Supplementary Figure 1–4 — The plots of the effect of coffee consumption GWMA on erectile dysfunction (Bovijn datasets). [file DataSheet1.zip › Supplementary information/Supplementary Figure/FigureS22.tif]

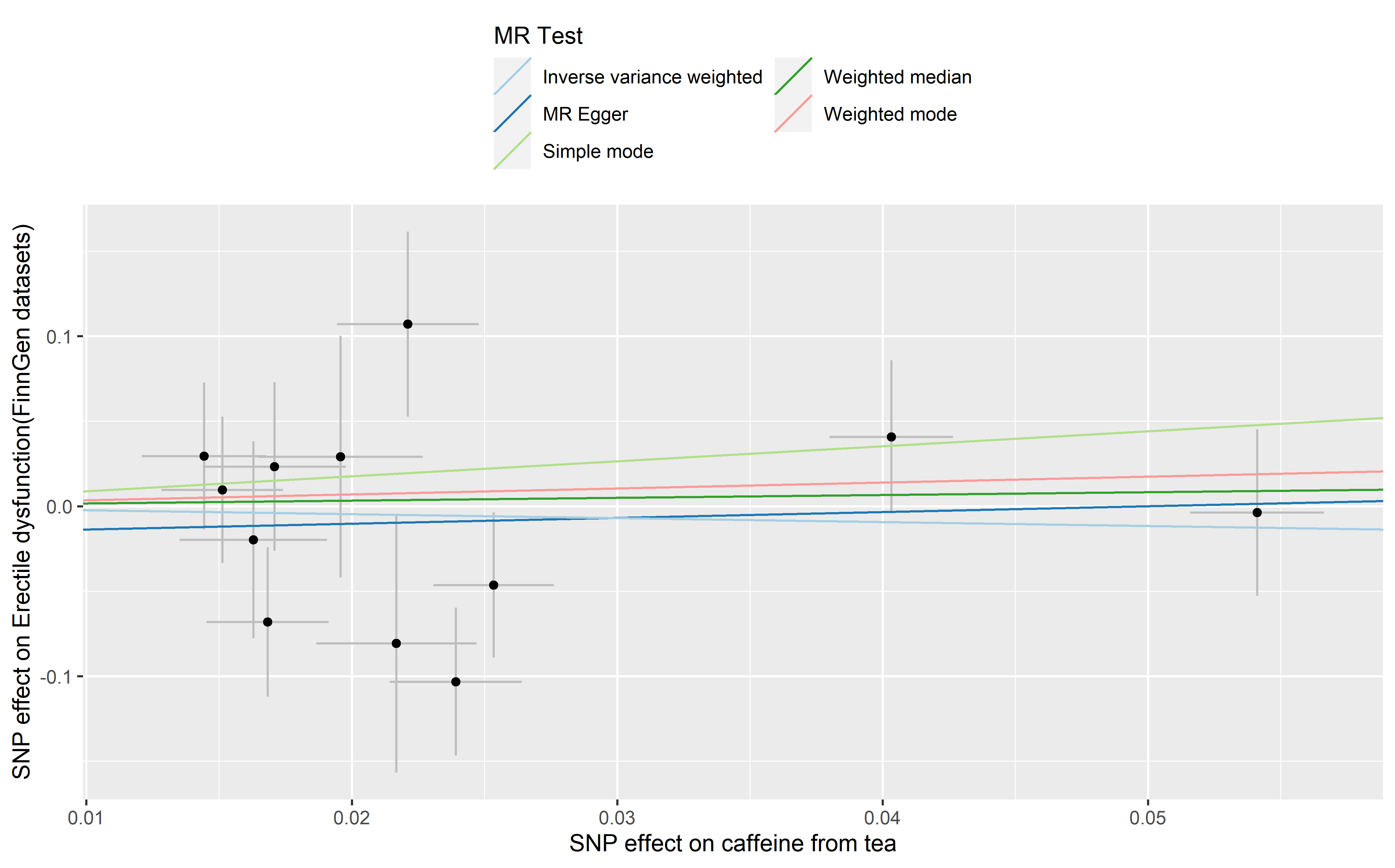

Supplement: Supplementary Figure 1–4 — The plots of the effect of coffee consumption GWMA on erectile dysfunction (Bovijn datasets). [file DataSheet1.zip › Supplementary information/Supplementary Figure/FigureS23.tif]

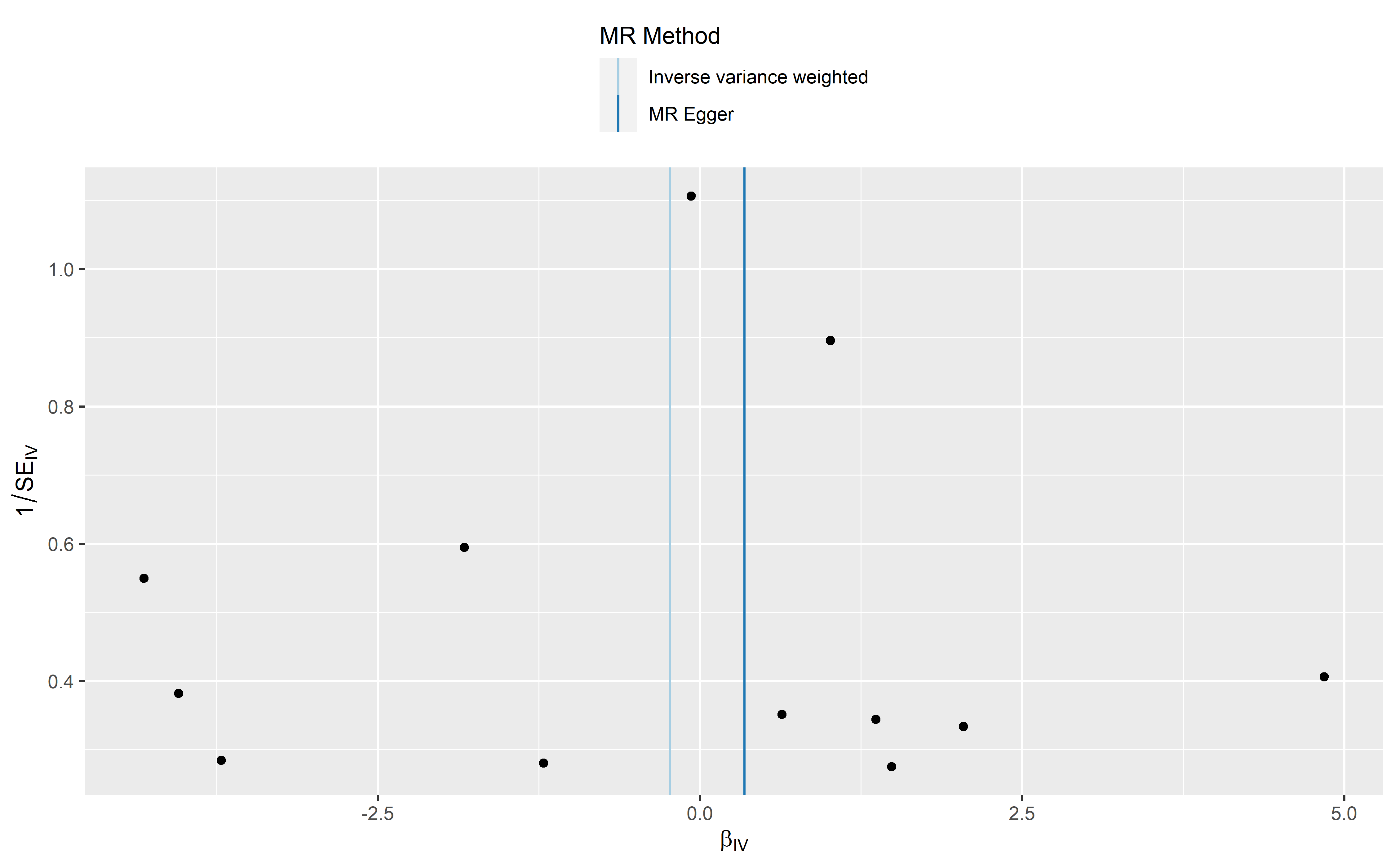

Supplement: Supplementary Figure 1–4 — The plots of the effect of coffee consumption GWMA on erectile dysfunction (Bovijn datasets). [file DataSheet1.zip › Supplementary information/Supplementary Figure/FigureS24.tif]

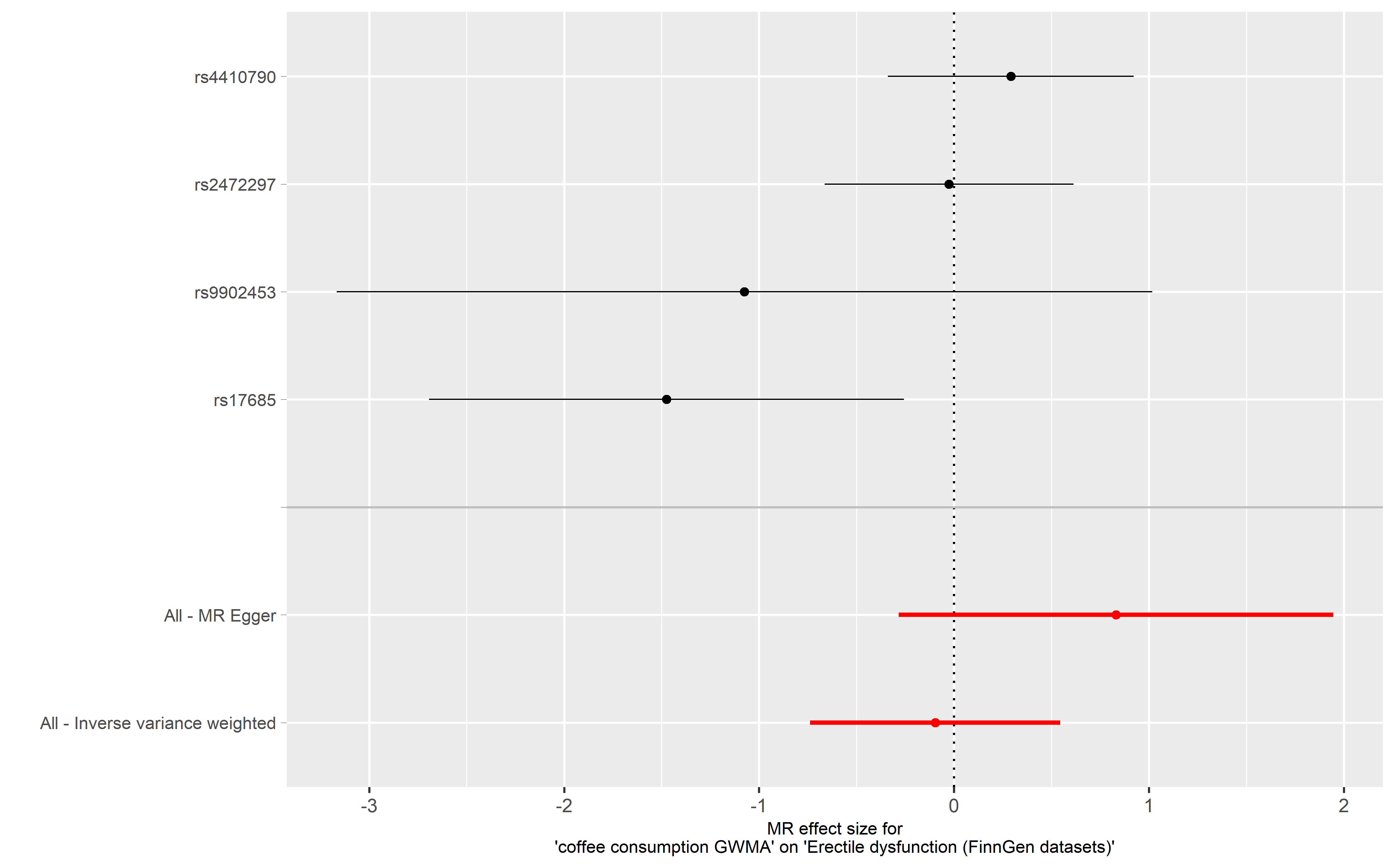

Supplement: Supplementary Figure 1–4 — The plots of the effect of coffee consumption GWMA on erectile dysfunction (Bovijn datasets). [file DataSheet1.zip › Supplementary information/Supplementary Figure/FigureS5.tif]

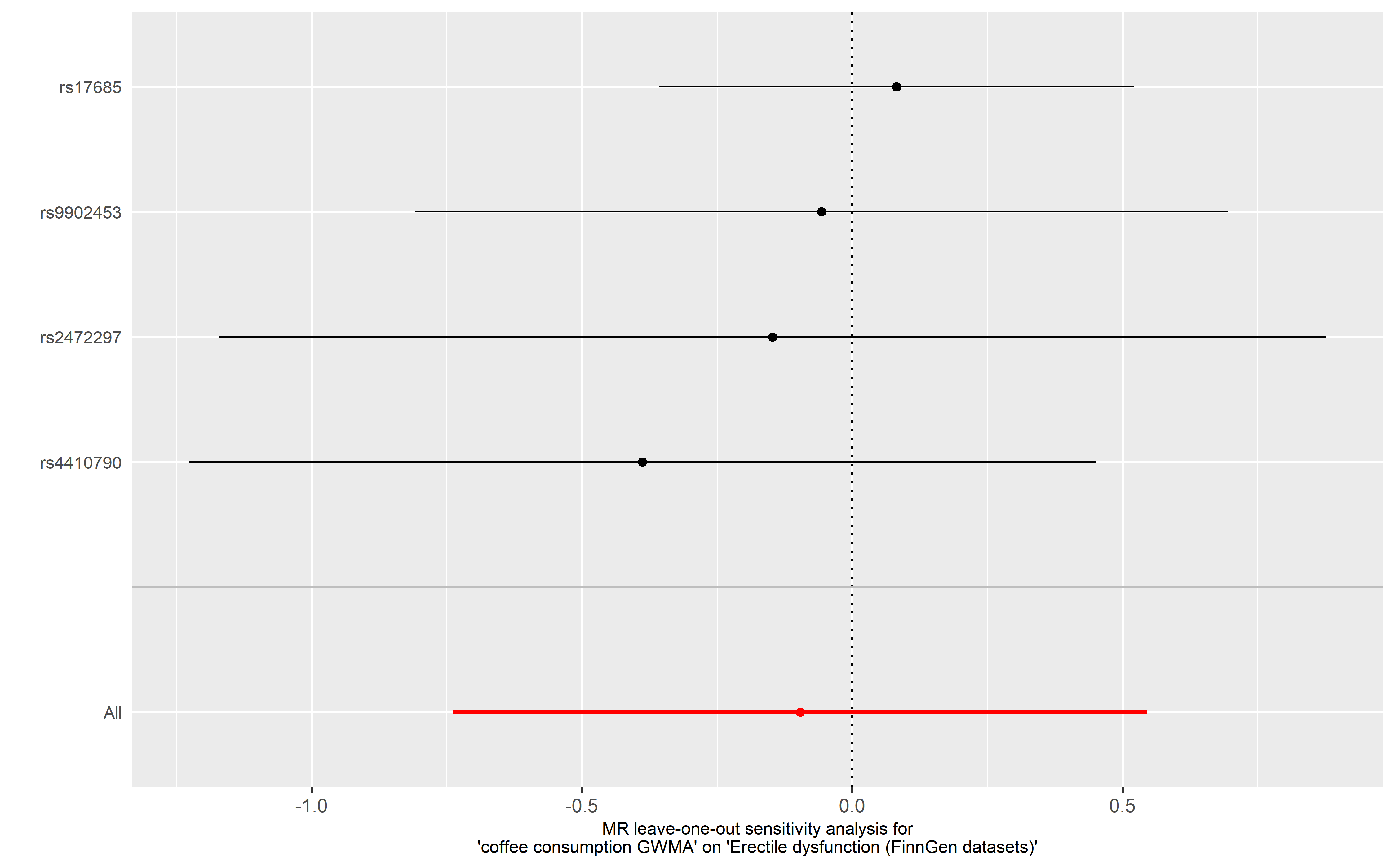

Supplement: Supplementary Figure 1–4 — The plots of the effect of coffee consumption GWMA on erectile dysfunction (Bovijn datasets). [file DataSheet1.zip › Supplementary information/Supplementary Figure/FigureS6.tif]

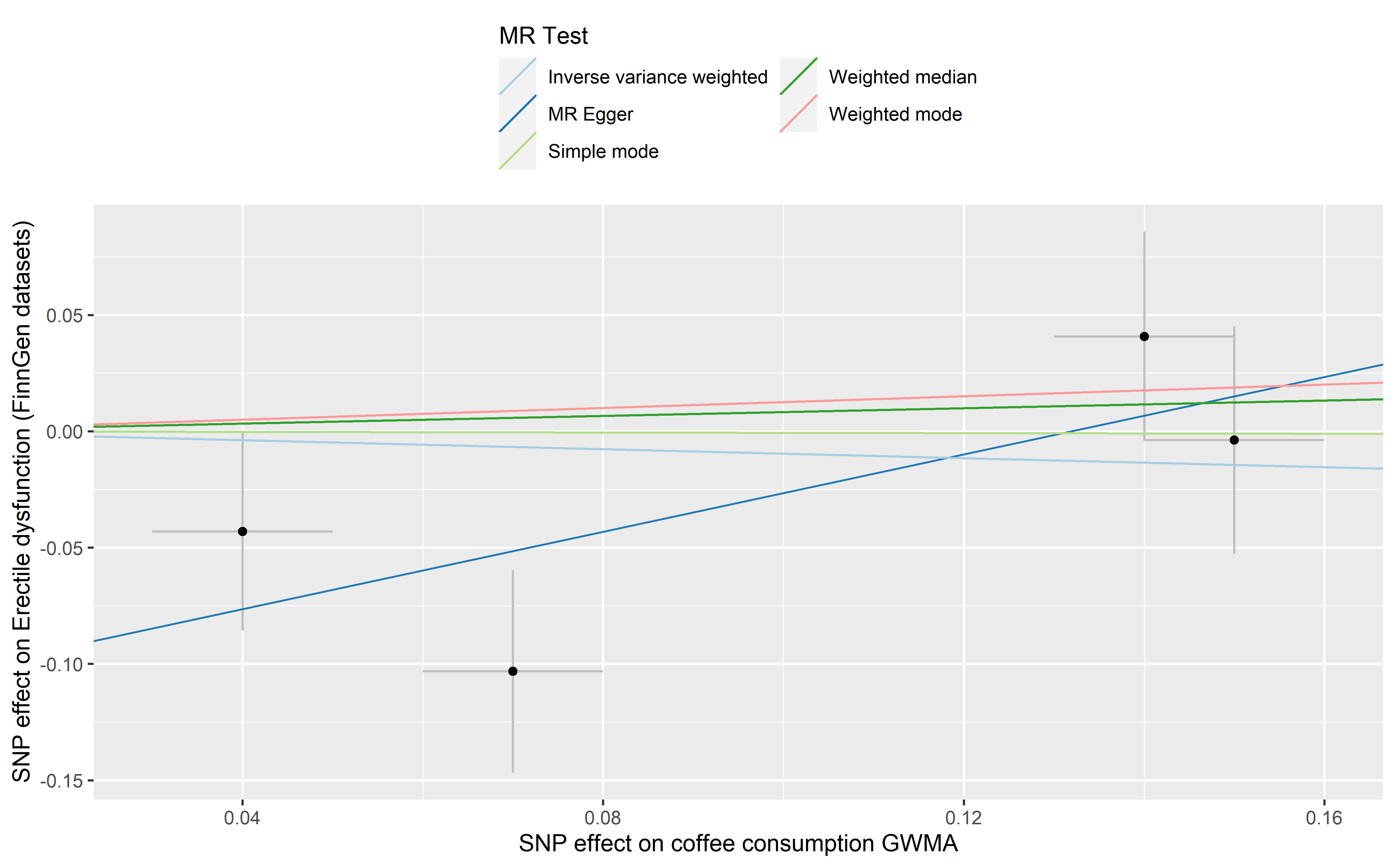

Supplement: Supplementary Figure 1–4 — The plots of the effect of coffee consumption GWMA on erectile dysfunction (Bovijn datasets). [file DataSheet1.zip › Supplementary information/Supplementary Figure/FigureS7.tif]

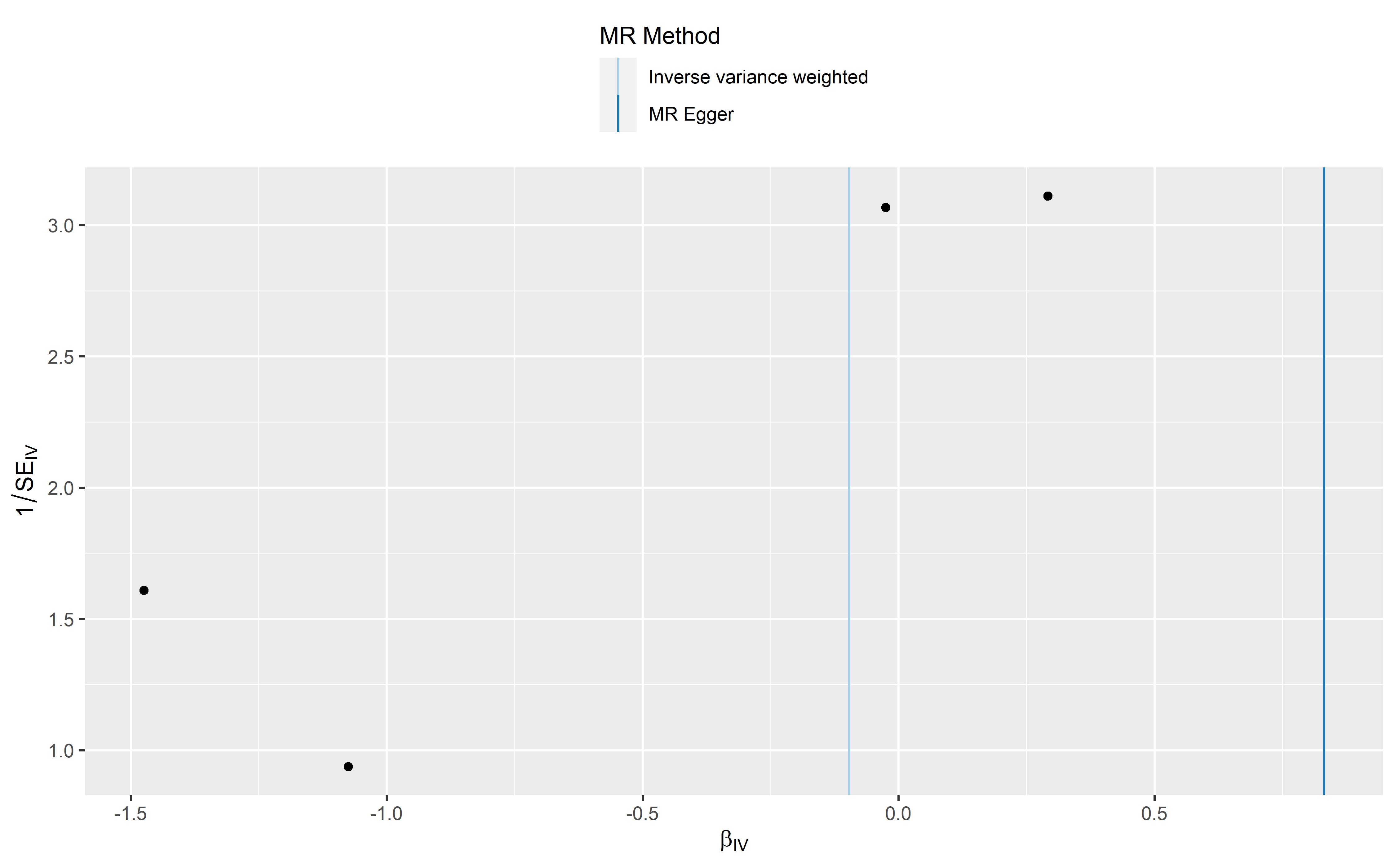

Supplement: Supplementary Figure 1–4 — The plots of the effect of coffee consumption GWMA on erectile dysfunction (Bovijn datasets). [file DataSheet1.zip › Supplementary information/Supplementary Figure/FigureS8.tif]
