# Supplementary material for: Potential genetic association between coffee/caffeine consumption and erectile dysfunction: a Mendelian randomization study and meta-analysis
Source: Front Endocrinol (Lausanne). 2024 Oct 9;15:1400491. doi: 10.3389/fendo.2024.1400491 (PMC11497819; doi:10.3389/fendo.2024.1400491)
Supplement: Supplementary Figure 1–4 — The plots of the effect of coffee consumption GWMA on erectile dysfunction (Bovijn datasets). [file DataSheet1.zip › Supplementary information/Supplementary Table1.doc]

Supplementary Table 1. Associations of SNPs with coffee/caffeine consumption and erectile dysfunction (Bovijn datasets).

| SNP | chr | pos | EA | OA | R^2^ | F | Exposure | | |  | Outcome | | |
| --- | --- | --- | --- | --- | --- | --- | --- | --- | --- | --- | --- | --- | --- |
|  |  |  |  |  |  |  | Beta | SE | *P* |  | Beta | SE | *P* |
| Coffee Consumption GWMA | | | | | | | | | | | | | |
| rs17685 | 7 | 75616105 | A | G | 0.0575% | 48.9988 | 0.0700 | 0.0100 | 9.06E-14 |  | 0.0093 | 0.0214 | 0.6658 |
| rs2472297 | 15 | 75027880 | T | C | 0.2561% | 224.9949 | 0.1500 | 0.0100 | 6.45E-47 |  | 0.0178 | 0.0224 | 0.4275 |
| rs4410790 | 7 | 17284577 | C | T | 0.2265% | 195.9955 | -0.1400 | 0.0100 | 1.48E-57 |  | 0.0131 | 0.0201 | 0.5140 |
| Coffee Consumption GWAS | | | | | | | | | | | | | |
| rs10127720 | 1 | 96273989 | C | T | 0.0113% | 42.6330 | 0.0111 | 0.0017 | 3.58E-11 |  | -0.0119 | 0.0221 | 0.5885 |
| rs1057868 | 7 | 75615006 | T | C | 0.0395% | 148.5344 | 0.0195 | 0.0016 | 5.26E-33 |  | 0.0071 | 0.0213 | 0.7398 |
| rs10997940 | 10 | 69886950 | C | T | 0.0082% | 30.6176 | 0.0083 | 0.0015 | 3.74E-08 |  | 0.0240 | 0.0200 | 0.2289 |
| rs13054099 | 22 | 41215672 | T | C | 0.0080% | 29.9272 | 0.0093 | 0.0017 | 3.17E-08 |  | -0.0261 | 0.0228 | 0.2521 |
| rs17687539 | 7 | 89662674 | A | G | 0.0082% | 30.8640 | 0.0100 | 0.0018 | 3.41E-08 |  | -0.0062 | 0.0241 | 0.7963 |
| rs1956218 | 14 | 33075243 | G | A | 0.0080% | 29.8843 | 0.0082 | 0.0015 | 3.62E-08 |  | 0.0060 | 0.0195 | 0.7579 |
| rs2297508 | 17 | 17715317 | G | C | 0.0096% | 35.9998 | 0.0090 | 0.0015 | 5.40E-09 |  | 0.0045 | 0.0200 | 0.8236 |
| rs2330783 | 22 | 24747031 | G | T | 0.0132% | 49.4452 | 0.0443 | 0.0063 | 1.57E-12 |  | 0.0306 | 0.0932 | 0.7427 |
| rs2465037 | 6 | 51179260 | C | A | 0.0102% | 38.2850 | 0.0099 | 0.0016 | 1.93E-10 |  | 0.0391 | 0.0206 | 0.0575 |
| rs2472297 | 15 | 75027880 | T | C | 0.1812% | 682.1279 | 0.0444 | 0.0017 | 5.19E-155 |  | 0.0178 | 0.0224 | 0.4275 |
| rs2597979 | 12 | 11189966 | G | C | 0.0091% | 34.1300 | 0.0111 | 0.0019 | 2.85E-09 |  | -0.0098 | 0.0250 | 0.6942 |
| rs2613458 | 11 | 1.15E+08 | C | A | 0.0092% | 34.5154 | 0.0094 | 0.0016 | 1.19E-08 |  | -0.0006 | 0.0211 | 0.9766 |
| rs4410790 | 7 | 17284577 | C | T | 0.1759% | 662.2009 | 0.0386 | 0.0015 | 5.59E-141 |  | -0.0131 | 0.0201 | 0.5140 |
| rs56113850 | 19 | 41353107 | C | T | 0.0165% | 61.8841 | 0.0118 | 0.0015 | 2.97E-15 |  | 0.0276 | 0.0206 | 0.1798 |
| rs57918684 | 17 | 60150383 | A | G | 0.0109% | 40.9598 | 0.0128 | 0.0020 | 2.99E-10 |  | 0.0217 | 0.0264 | 0.4101 |
| rs586346 | 6 | 31875712 | T | C | 0.0107% | 40.1109 | 0.0095 | 0.0015 | 5.98E-10 |  | 0.0022 | 0.0206 | 0.9148 |
| rs597045 | 11 | 56272114 | A | T | 0.0117% | 43.8904 | 0.0106 | 0.0016 | 6.62E-11 |  | 0.0293 | 0.0211 | 0.1640 |
| rs6062682 | 20 | 62891820 | T | C | 0.0116% | 43.5598 | 0.0099 | 0.0015 | 3.16E-11 |  | 0.0034 | 0.0197 | 0.8643 |
| rs6124969 | 20 | 45816277 | T | C | 0.0092% | 34.4176 | 0.0088 | 0.0015 | 1.06E-08 |  | -0.0130 | 0.0200 | 0.5171 |
| rs75347775 | 19 | 18495908 | A | G | 0.0085% | 31.8891 | 0.0096 | 0.0017 | 2.28E-08 |  | 0.0283 | 0.0227 | 0.2134 |
| rs8056750 | 16 | 70927078 | T | C | 0.0079% | 29.5662 | 0.0087 | 0.0016 | 4.71E-08 |  | 0.0004 | 0.0210 | 0.9839 |
| rs993885 | 5 | 7379003 | G | A | 0.0094% | 35.2043 | 0.0089 | 0.0015 | 7.34E-09 |  | -0.0111 | 0.0203 | 0.5842 |
| Caffeine From Tea | | | | | | | | | | | | | |
| rs11204711 | 1 | 150682115 | A | G | 0.0097% | 38.1979 | -0.0144 | 0.0023 | 6.40E-10 |  | 0.0158 | 0.0293 | 0.5889 |
| rs12591786 | 15 | 60902512 | C | T | 0.0100% | 39.6460 | 0.0196 | 0.0031 | 3.00E-10 |  | 0.0664 | 0.0271 | 0.0142 |
| rs132919 | 22 | 41809903 | G | C | 0.0101% | 40.1094 | -0.0171 | 0.0027 | 2.40E-10 |  | 0.0081 | 0.0353 | 0.8178 |
| rs140775622 | 20 | 62962869 | C | T | 0.0126% | 49.7315 | -0.0226 | 0.0032 | 1.80E-12 |  | 0.0360 | 0.0722 | 0.6174 |
| rs17685 | 7 | 75616105 | G | A | 0.0233% | 92.3713 | -0.0239 | 0.0025 | 7.20E-22 |  | -0.0093 | 0.0214 | 0.6658 |
| rs192084998 | 5 | 152077481 | G | A | 0.0105% | 41.5006 | 0.0158 | 0.0025 | 1.20E-10 |  | -0.0292 | 0.0437 | 0.5038 |
| rs2117137 | 3 | 89525505 | A | G | 0.0089% | 35.2570 | -0.0135 | 0.0023 | 2.90E-09 |  | -0.0182 | 0.0198 | 0.3594 |
| rs2465018 | 6 | 51241140 | G | A | 0.0174% | 68.7786 | -0.0221 | 0.0027 | 1.10E-16 |  | 0.0436 | 0.0350 | 0.2129 |
| rs2472297 | 15 | 75027880 | C | T | 0.1163% | 460.7560 | -0.0541 | 0.0025 | 3.30E-102 |  | -0.0178 | 0.0224 | 0.4275 |
| rs4410790 | 7 | 17284577 | T | C | 0.0763% | 302.1771 | -0.0403 | 0.0023 | 1.10E-67 |  | 0.0131 | 0.0201 | 0.5140 |
| rs4817505 | 21 | 34343828 | T | C | 0.0110% | 43.5133 | -0.0151 | 0.0023 | 4.20E-11 |  | -0.0038 | 0.0199 | 0.8472 |
| rs56188862 | 1 | 174189269 | T | C | 0.0136% | 53.6616 | 0.0168 | 0.0023 | 2.40E-13 |  | -0.0091 | 0.0200 | 0.6480 |
| rs62534435 | 9 | 7042938 | C | G | 0.0087% | 34.4349 | -0.0163 | 0.0028 | 4.40E-09 |  | -0.0312 | 0.0246 | 0.2056 |
| rs73053413 | 12 | 11329548 | C | T | 0.0130% | 51.5078 | 0.0217 | 0.0030 | 7.10E-13 |  | 0.0048 | 0.0273 | 0.8594 |
| rs9624470 | 22 | 24820268 | G | A | 0.0314% | 124.4245 | -0.0253 | 0.0023 | 6.80E-29 |  | 0.0143 | 0.0198 | 0.4693 |

R^2^ indicates the proportion of variance explained by each of the SNPs: 𝑅^2^ = [2 × Beta^2^ × (1 - EAF) × EAF]/[2 × Beta^2^ × (1 - EAF) × EAF + 2 × SE^2^ × 𝑁 × (1 - EAF) × EAF]; F-statistic for each of SNPs was calculated using the formula: R^2^ × (𝑁 - 2) / (1 - R^2^). SNP, single nucleotide polymorphisms; EA, effect allele; OA, other allele; Beta, genetic effects of SNP on prescription opioid use; SE, standard error; GWMA: genome-wide meta-analysis; GWAS: genome-wide association study.
